# Day-to-day dynamics of fetal heart rate variability to detect chorioamnionitis in preterm premature rupture of membranes

**DOI:** 10.1371/journal.pone.0305875

**Published:** 2025-01-02

**Authors:** Aline Taoum, Guy Carrault, Caroline Tesson, Maxime Esvan, Bruno Laviolle, Linda Lassel

**Affiliations:** 1 LTSI, Université de Rennes, INSERM, Rennes, France; 2 CHU Rennes, Inserm, CIC 1414 (Centre d’Investigation Clinique de Rennes), Rennes, France; 3 CHU Rennes, Rennes, France; Universita Politecnica delle Marche, ITALY

## Abstract

**Background:**

Chorioamnionitis is recognized as a major consequence of preterm premature rupture of membranes (PPROM), and a frequent cause of neonatal morbidity and mortality. The association between fetal heart rate (FHR) and chorioamnionitis remains unclear.

**Objectives:**

The aim of this study was to evaluate the dynamics of FHR in a PPROM population at the approach of delivery according to the presence or absence of chorioamnionitis.

**Materials & methods:**

120 pregnant women with PPROM between 26 and 34 weeks’ gestation were enrolled in this multicenter prospective unblinded study. All participants were fully informed of the study’s objectives. 39 of the 120 patients were included in the analysis of FHR recordings. The analysis consisted of extracting features from computerized FHR analysis (cFHR) and fetal heart rate variability analysis (FHRV) in the temporal, frequency and nonlinear domains. Then, each set of features was analyzed separately using the multiple factor analysis, where three groups were defined as the feature set for days 0, -1 and -2 prior to birth. The distances between the global projection and the projections for each day were computed and used in the ROC analysis to distinguish chorioamnionitis from non-chorioamnionitis group.

**Results:**

The results showed that there were significant differences in certain features between populations with and without chorioamnionitis. The distinction between the two populations reached an area under the curve (AUC) of only 37% [34–40] for cFHR features and 63% [59–66] for time-domain FHRV features when comparing all stages of chorioamnionitis to non-chorioamnionitis subjects. When only stage 3 chorioamnionitis was compared to non-chorioamnionitis patients, the AUC reached 90% [88–93] for nonlinear-domain and 84% [82–87] for time-domain FHRV features, whereas it was limited to 71% [68–74] using cFHR features.

**Conclusion:**

The present study suggests that the HRV features are more reliable for diagnosing chorioamnionitis than cFHR, and that the assessment of features dynamics over several days is an interesting tool for detecting chorioamnionitis. Further study should be carried out on a larger sample to confirm these findings, improve the diagnostic performance of chorioamnionitis and help clinicians decide on delivery criteria.

## Introduction

Preterm premature rupture of membranes (PPROM) refers to the rupture of fetal membranes prior to 37 gestational weeks (GW) [1]. It occurs in 2–3% of pregnancies and is responsible for one-third of preterm deliveries and around 20% of perinatal mortality [1, 2]. The main risk factors for PPROM are a history of premature delivery, preconceptional cervical abnormalities, vaginal bleeding, cervical shortening during pregnancy, genital infections and intrauterine infection. However, in most cases, PPROM occurs in the absence of any risk factor [1, 3]. In addition to prematurity, PPROM exposes the fetus and its adnexa to serious complications, including placental abruption, umbilical cord compression and intrauterine infection, also known as chorioamnionitis [3].

The onset of chorioamnionitis significantly increases the risk of neonatal complications, including perinatal death, early neonatal sepsis, pneumonia, and long-term disability [4, 5]. The diagnosis of chorioamnionitis is based primarily on clinical symptoms, with a combination of maternal or fetal tachycardia, maternal fever, uterine tenderness or elevated white blood cell count. However, the presence of one or more of these signs does not necessarily confirm the presence of chorioamnionitis. Amniocentesis may suggest or confirm prenatal diagnosis, but is not considered a standard of care in cases of PPROM because it is a risky procedure [6, 7]. Anatomopathological tests are often conclusive in the diagnosis of chorioamnionitis, but are performed after the infant is delivered. It is therefore crucial to develop an early marker of chorioamnionitis that is specific, non-invasive, and easily accessible in routine clinical practice, in order to improve the neonatal prognosis of PPROM. More specifically, it would enable a rapid and reliable online analysis to predict chorioamnionitis and help clinicians make a decision on delivery criteria.

In obstetrics, electronic fetal monitoring (EFM) or cardiotocography (CTG) has been widely used to monitor uterine contractions and fetal heart rate (FHR) with the aim of assessing fetal well-being during the intrapartum period [8]. FHR assessment has been shown to be effective in reducing the risk of preventable intrapartum fetal death, and in alerting to a large proportion of neonatal complications such as fetal hypoxia, fetal acidemia, neonatal encephalopathy, and cerebral palsy [9]. However, studies carried out on chorioamnionitis have shown contradictory results or an absence of association between FHR and chorioamnionitis [10–15]. The FHR reflects the behavior of the cardiovascular system and is modulated by the fetal brain and its autonomous nervous system (ANS) [16]. The variability of the FHR (FHRV) is correlated with ANS function via sympathetic and parasympathetic tones. Studies in the literature have shown the importance of assessing HRV to detect infections in adults and infants, and particularly premature babies [17–19]. The study of FHRV features therefore appears to be a potential, as yet unexploited, avenue of research for the early detection of chorioamnionitis. It could enable a non-invasive approach to biological rhythms, offering a new diagnostic and/or prognostic approach.

The aim of our study is to observe the evolution of the features of the FHR in a population of PPROM approaching delivery, according to the presence or absence of chorioamnionitis. After recording and preprocessing FHR data, we extracted features directly from the computerized FHR recordings (cFHR) and features from the HRV analysis in the time, frequency and nonlinear domains. We were interested in observing the features dynamics over the last three days of the PPROM latency period (last 72 hours before birth) to better characterize the period of onset of chorioamnionitis. The preceding days were not taken into account, as the presence of chorioamnioninis is not certain. Multiple factor analysis (MFA) was used to study each set of features described by the three days before birth. MFA provides partial projections from the features set of each day separately and a global projection from the concatenation of the features of all days. ROC analyses were then performed on the distances computed between the partial and global projections to distinguish subjects with chorioamnionitis from those without. To our knowledge, there is no equivalent study in the literature.

## Materials and methods

**Fig 1:**
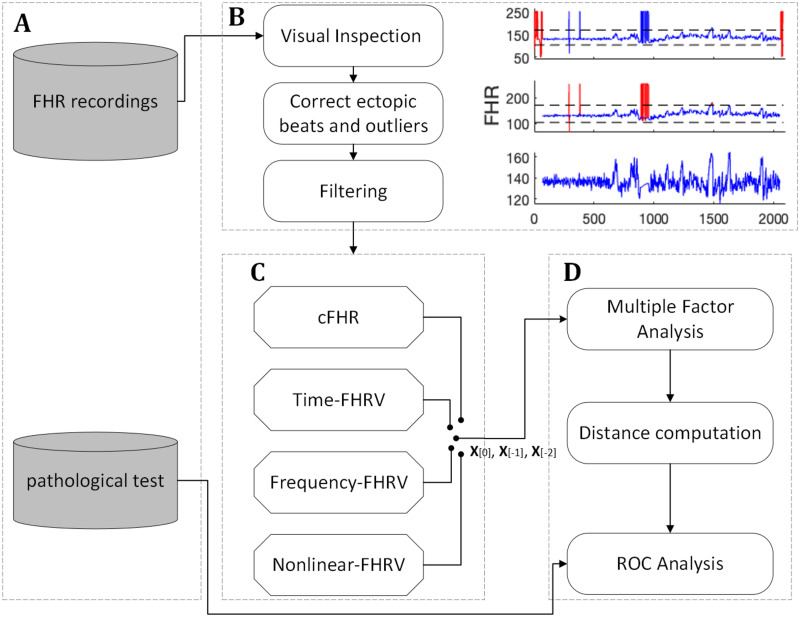
Flowchart diagram of the processing method.

In the following paragraphs, we describe the study design, the enrolled population, the data recorded, the preprocessing and the data analysis procedure. The whole pipeline is visualized in Fig 1 and described in the following sections. We have used the TREND reporting guidelines and provided in S1 Checklist [20].

### Study population and design

This is a multicenter prospective case-control study led by Rennes University Hospital (CHU of Rennes) to characterize the pattern of FHR based on the presence or not of chorioamnionitis in a population of PPROM (clinical trial number NCT02901795). It was conducted in accordance with the requirements of clinical practice in Europe, French public health code and the ethical principles of Helsinki declarations. Patients were orally and fully informed of the objectives of the study, of their right to refuse to participate, and of the possibility of withdrawing consent at any time, including for their newborns. All this details were included in an information and no-objection letter given to patients. Patients gave verbal informed consent, and if they objected to participate in the study, they were asked to submit a written refusal. The study was approved by the local research Ethics Review committee of the CHU of Rennes (Notice number 14.76). The study protocol of the clinical trial can be found in S1 and S2 Files, for French and English versions, respectively.

A pilot study by the CHU of Rennes was performed and suggested that a sample of 60 analyzable patients has a power of 95% to detect significant difference between the chorioamnionitis and non-chorioamnionitis groups. Given that 50% of PPROMs deliver within 24–48 hours after the rupture [6], a sample of 120 patients was required. Thus, 120 pregnant women were enrolled in 4 French hospitals located in Rennes, Angers, Nantes and Poitiers between 09 December 2014 to 16 December 2021. All participants were adult with a singleton pregnancy hospitalized for PPROM between 26 and 34 GW. Exclusion criteria were: pregnant women with pre-existing or gestational diabetes, multifetal gestation, fetal malformation on ante/post-natal examination, active maternal smoking, neonatal hypotrophy (birth weight <10^th^ AUDIPOG percentile), and presence of maternal pathology (heart disease, pulmonary embolism, hypertension, chronic renal failure, chronic obstructive pulmonary disease or autoimmune disease).

PPROM was diagnosed either by frank discharge of amniotic fluid during speculum examination, or by a vaginal diagnostic test for Insulin Growth factor Binding Protein 1 (IGFBP-1) in case of doubt on clinical examination. According to French guidelines for PPROM, all patients received nifedipine or atosiban for tocolysis, amoxicillin intravenous injections as antibiotic prophylaxis and betamethasone injections as corticosteroid therapy [1, 21]. The name of the medicine, the method of administration, the dates and duration of treatment were collected. All patients with PPROM were closely monitored from the onset of PPROM until after birth. During the monitoring period, two types of data were collected from patients: clinical and physiological data.

- Clinical data: Maternal clinical data were collected throughout the surveillance period and included demographic data, precise pregnancy dating (obstetrical ultrasound performed between 11 GW + 1 day and 14 GW + 6 days), obstetric history, general medical and surgical history, date of PPROM in GW, color of amniotic fluid, type, date and duration of each treatment the participant was receiving. Clinical data relating to delivery included information on mode and reason of delivery (if suspicion of clinical chorioamnionitis), context of onset of labor (maternal fever, changes in amniotic fluid, uterine contractions, abnormalities in FHR), mode of delivery. Finally, neonatal clinical data included birth weight, pH and lactate of umbilical cord and, in case of neonatal death, date, time and result of autopsy if performed, as well as the result of the anatomopathological examination of the placenta, if performed. Pathological examination was used to determine the stage of chorioamnionitis infection, where stage 0 indicating that there is no infection and stages 1, 2 and 3 indicating the presence of chorioamnionitis and the stage of infection.
- Physiological data: These included FHR recordings during the follow-up period. FHR recording were carried out using an F3 Fetal Monitor cardiotocograph (EDAN Instruments, Inc.) / Sonicaid Oxford 8002 system. This device has the advantage of a 60-hour internal memory, enabling recordings to be stored as independent TRC (Trace File) files, which can be transferred to a computer via a USB port. Ideally, two recordings per day (every 12 h) were performed for each patient for 30 min from the date of membrane rupture to delivery. All FHR recordings had to meet Dawes and Redan criteria or last at least 30 min. This routine was not always respected, i.e. sometimes a single FHR or no FHR was recorded for several days, and conversely, in other cases several FHR were recorded per day.

The data was accessed for research purposes on November 2022. Only one of the authors had access to information that could identify individual participants in one of the centers, by virtue of her role as the healthcare professional responsible of the data collection in the CHU of Rennes.

### Data preprocessing

Processing was done with respect of the french application of EU regulation related to protection of personal data, data processing, data files and individual liberties. All data were pseudonymized after monitoring by the medical staff.

The FHR recordings were preprocessed to correct for outliers and ectopic beats, as shown in Fig 1B. First, All FHR data were visually inspected to identify and eliminate periods with false heart rate values at the beginning and end of the recordings. Next, all ectopic beats and outliers were identified and corrected. Ectopic beats are defined as any beat that differs from the previous one by more than 8 × 75^th^ percentile of values, or by more than 25 beats per minute (bpm). All the values that lies between two consecutive ectopic beats of opposite directions are considered an outlier period. Outlier beats are all beats with a value > 1.2 x 75^th^ percentile of values or a value < 0.8 x 25^th^ percentile of values. Periods with ectopic or outlier values are replaced by spline interpolation if they last less than 30 s, otherwise they are considered periods of data loss and replaced by blanks. No corrections were made during the period of data loss, in order to preserve as much of the variation in the original data as possible. Finally, recordings are filtered using a zero-phase digital filtering to eliminate baseline drift.

### Features extraction

A range of features were extracted based on the computerized analysis of the FHR and the heart rate variability (HRV) analysis using custom made Matlab codes (Fig 1C). FHR recordings were transformed into RR-intervals before extracting HRV features. All features, further detailed in the following paragraphs, were calculated for each measurement session. A list of the extracted features can be found in Table 1.

**Table 1:** Summary of the extracted features.

Type	Name
cFHR	baseline FHR, Accelerations (number/minute, average duration, cumulative size/minute), Deceleration (number/minute, average duration, cumulative size/minute), LTV, STV, Episodes of high variation (number/minute, duration/min), Episodes of low variation (number/minute, duration/min)
Time-FHRV	Mean, SD, maximum, minimum, RMSSD, SDSD, skewness, kurtosis
Frequency-FHRV	VLF, LF, HF, LF/HF, LFnu, HFnu, Total power
Nonlinear-FHRV	Approximate entropy, sample entropy, SD1 and SD2 from poincaré plot, short and long term correlations from DFA, acceleration and deceleration capacities

#### Computerized analysis of FHR (cFHR)

The features follows the definitions of Jones et al. [22]. They include the baseline FHR in beats per minute (bpm), accelerations and deceleration, long and short term variations, and episodes of low and high FHR variability [22]. Accelerations are defined as an increase in the FHR of 10 or 15 bpm for more than 15 s. Decelerations are defined as a decrease in FHR of 10 bpm for more than 60 s, or of 20 bpm for more than 30 s. Accelerations and decelerations were presented by their relative number per minute, their average durations, and their relative cumulative sizes over the recordings. Long-term variation (LTV) over each minute is the difference in ms between the highest and lowest value of 16 sections of the minute. The overall LTV is the average of the LTV computed for each minute. The short-term variation (STV) corresponds to the measurement of micro-fluctuations in the FHR. It is computed as the 1/16 min epoch-to-epoch variation averaged over each minute and over the whole recording. Episodes of high (low) variation are any part of the recording where the variation over one minute is greater than 32 ms (less than 30 ms, respectively) for at least 5 of 6 consecutive minutes. These episodes are characterized by their relative number, and relative durations.

#### Time-domain HRV analysis (time-FHRV)

From RR intervals, we computed the mean, standard deviation (SD), maximum, minimum, root mean square of successive differences (RMSSD), standard deviation of successive differences (SDSD), skewness, and kurtosis [23].

#### Frequency-domain HRV analysis (Frequency-FHRV)

HRV analysis in the spectral domain aims to describe the power distribution (power spectral density—PSD) of RR intervals over frequency bands. The Lomb-Scargle method waas used to estimate PSD because RR intervals are in nature unevenly sampled data and they may contain missing data due to removed long outlier periods [24]. Frequency-domain features included very low frequency power (VLF: 0.001–0.02 Hz), low-frequency power (LF: 0.02–0.2 Hz), high-frequency power (HF: 0.2–1.5 Hz), the ratio of low-frequency power to high-frequency power (LF/HF), normalized low-frequency power (LFnu = LF/(LF + HF)), normalized high-frequency power (HFnu = HF/ (LF + HF)), and total power calculated over all frequency bands (TtlPwr = VLF + LF + HF) [23].

#### Non-linear HRV analysis (nonlinear-FHRV)

They are used to describe the complexity and the unpredictability of RR intervals, which result from the complex interactions of the many mechanisms modulating the cardiac variability. These parameters are the approximate entropy (ApEn), sample entropy (SampEn), the short and long term fluctuation indexes (SD1 and SD2) from the Poincaré plot, short and long term correlations from the detrended fluctuation analysis, and the acceleration and deceleration capacities [23, 25].

### Multiple Factor Analysis

Multiple Factor Analysis (MFA) analyzes a set of observations described by several groups of variables. The analysis provides an integrated picture of the observations and the relationships between the groups of variables [26]. In this particular study, we found it interesting to investigate the relationships and thus the dynamics between the last three days of recordings before birth for a given type of features (i.e. cFHR, time-FHRV, frequency-FHRV and nonlinearFHRV). The originality of MFA lies in the fact that it allows to study the impact of groups of variables on an observation by simultaneously visualizing the observation described by all the variables (global projection) and by each group of variables (partial projection).

For each features type, the features are sorted in three matrices ***X***^[0]^, ***X***^[−1]^, ***X***^[−2]^ of size (*N*, *M*) each, denoting the datasets at the days 0, -1, and -2. Days 0, -1, and -2 correspond, respectively, to the last 24 hours before birth, 24 to 48 hours before birth and 48 to 72 hours before birth. *N* = 39 is the number of subjects, and *M* is the number of features in dataset ***X***^[*k*]^. In our study, *M*_*c*_ = 13, *M*_*t*_ = 8, *M*_*f*_ = 7, *M*_*nl*_ = 8, respectively, for computerized analysis of FHR, time-FHRV, frequence-FHRV and nonLinear-FHRV. If the subject has several recordings per day, the median value of these recordings is considered. Matrices were centered and normalized according to each column.

MFA consists of two steps as shown in Eq (1). First, a principal component analysis (PCA) is performed for each group of variables (datasets) and expressed via its singular value decomposition (SVD) [27]. The weights of each matrix are obtained by the inverse of the first squared singular value of its PCA and are used to normalize the datasets ***X***^[0]^, ***X***^[−1]^, ***X***^[−2]^. Second, the normalized datasets are combined all together to form a unique matrix ***X*** = [***X***^[0]^, ***X***^[−1]^, ***X***^[−2]^] and a global PCA is performed on ***X*** [28]. General factor scores and factor loadings are obtained as being the projections in the new principal components or dimensions of the observations and the variables, respectively. In addition, partial factor scores for each dataset ***X***^[*k*]^ can be obtained as its projection onto the global space.

$$\begin{array}{c} X=\begin{array}{c} \underset{↓}{\underset{︸}{\left[\begin{array}{ccc} X_{1}^{\left[0\right]}\cdots X_{M}^{\left[0\right]} & X_{1}^{\left[-1\right]}\cdots X_{M}^{\left[-1\right]} & X_{1}^{\left[-2\right]}\cdots X_{M}^{\left[-2\right]}\\ \underset{↓}{\underset{︸}{\left[\begin{array}{ccc} x_{1,1}^{0} & \cdots & x_{1,M}^{0}\\ \vdots & x_{n,m}^{0} & \vdots \\ x_{N,1}^{0} & \cdots & x_{N,M}^{0} \end{array}\right]}} & \underset{↓}{\underset{︸}{\left[\begin{array}{ccc} x_{1,1}^{-1} & \cdots & x_{1,M}^{-1}\\ \vdots & x_{n,m}^{-1} & \vdots \\ x_{N,1}^{-1} & \cdots & x_{N,M}^{-1} \end{array}\right]}} & \underset{↓}{\underset{︸}{\left[\begin{array}{ccc} x_{1,1}^{-2} & \cdots & x_{1,M}^{-2}\\ \vdots & x_{n,m}^{-2} & \vdots \\ x_{N,1}^{-2} & \cdots & x_{N,M}^{-2} \end{array}\right]}}\\ {\text{PCA}}^{\left[0\right]} & {\text{PCA}}^{\left[-1\right]} & {\text{PCA}}^{\left[-2\right]}\\ \times & \times & \times \\ {\text{weights}}^{\left[0\right]} & {\text{weights}}^{\left[-1\right]} & {\text{weights}}^{\left[-2\right]} \end{array}\right]}}\\ \text{Global}\;\text{PCA} \end{array} \end{array}$$ (1)

It is worth noting that the general factor scores matrix is the centroid or the barycenter of the partial factor scores [28]. The observations can be visualized in 2- or 3-dimensional spaces using the general factor scores alone or by adding the partial factor scores. The importance of each dimension is measured by its eigenvalue and its percentage of variance, i.e. the amount of information it carries. In addition, each dimension is influenced either by observations, variables, or group of variables. This influence is determined by the contribution of an observation, a variable or a set of variables to a dimension [28]. The analyses was performed using the FactoMineR package on RStudio [29].

### Distance computation

To better understand the variation in the data and take into account of the evolution over time, we calculated the distances between the partial factor scores and the barycenter or global factor scores of the MFA. This enables us to summarize the dispersion between groups of variables (days before birth) for a given type of parameter. Distances are calculated on the first 4 principal dimensions. It is defined by *d*_1−4_, where *d* is the 4-dimensional Euclidean distance function.

### Statistical analysis

Parameters are expressed as mean ± standard deviation. A Kolmogorov-Smirnov or Mann-Whitney statistical test was performed, as appropriate, to compare data. A p-value < 0.05 was considered statistically significant.

ROC analysis was performed on the computed distances to distinguish between subjects with and without chorioamnionitis using RStudio. Non-chorioamnionitis subjects were all cases with a negative anatomopathological test (stage 0); whereas chorioamnionitis population included cases whose chorioamnionitis had been diagnosed at stages 1, 2 and 3. We performed the ROC analysis by first considering all the chorioamnionitis population, then considering stages 2 and 3 or only stage 3 subjects. The analysis is performed using the 5-fold cross-validation method repeated 10 times.

## Results

### Population characteristics

**Fig 2:**
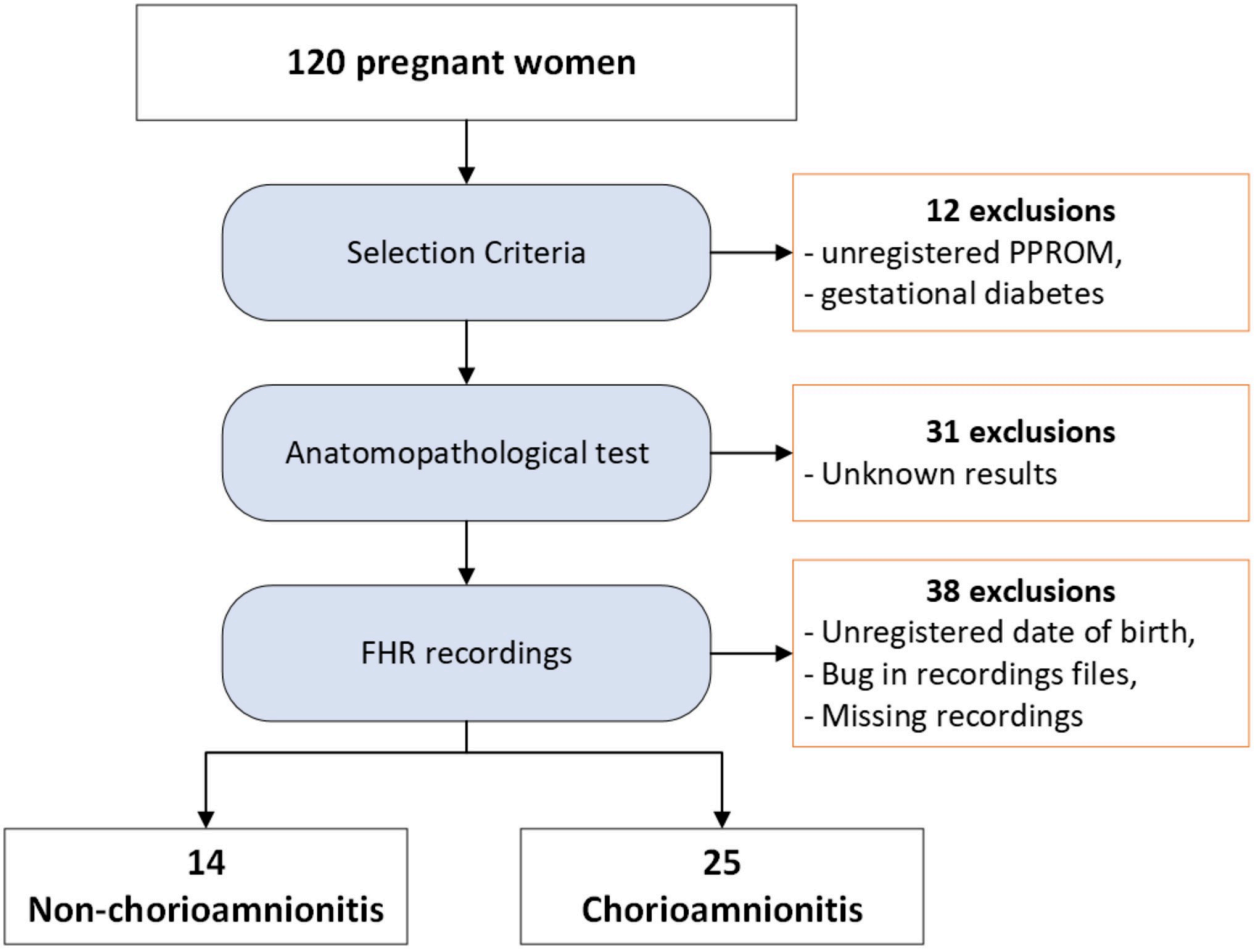
Inclusions and exclusions of patients.

The recruitment was performed from 09 December 2014 to 16 December 2021. Fig 2 shows patient exclusions throughout the analysis. Of the 120 pregnant women with PPROM initially included in the study, 12 were excluded at initial selection due to an unrecorded date of PPROM or the presence of gestational diabetes, 31 were excluded due to the absence of anatomopathological results, and 38 were excluded for one of the following reasons: 1) date of birth not recorded, 2) error in date and time of recordings due to a bug in the monitoring device, 3) No FHR recording for at least one day in the 3 days prior to delivery. Consequently, data from only 39 pregnant women were considered for the analysis, 25 pregnant women were diagnosed as chorioamionitis while 14 women were not.

The mean age of the enpatients was 30 ± 6 years. Table 2 summarizes the clinical characteristics of the 39 pregnant women included in this study. The features presenting the most significant differences between chorioamnionitis and non-chorioamnionitis populations are shown in Table 3 along with the p-value of the statistical test. The comparison for all features can be found in S1 Table.

**Table 2:** Clinical characteristics in cases of PPROM included in the study according to the result of the anatomopathological test.

	Non-Chorioamnionitis	Chorioamnionitis		
	Stage 0	Stage 1	Stage 2	Stage 3
Number	14	6	14	5
Maternal age (yo)	29 [28–33]	30 [25–36]	33 [31–34]	36 [34–37]
Maternal BMI	20 [19–23]	27 [24–28]	23 [20–24]	21 [20–23]
Date of PPROM (GA)	30 [27–31]	30 [29–30]	31 [28–33]	29 [25–30]
Date of delivery (GA)	32 [31–33]	32 [31–33]	32 [30–34]	33 [30–34]
Mode of delivery [C-section]	3 (21%)	0 (0%)	2 (14%)	2 (40%)
Suspicion of chorioamnionitis [Yes]	3 (21%)	3 (50%)	6 (43%)	4 (80%)
Birth weight (kg)	1.83 [1.66–2.01]	1.8 [1.49–2.02]	1.82 [1.5–1.93]	1.74 [1.66–1.85]
Percentile AUDIPROG	50 [41–56]	50 [50–55]	60 [40–64]	47 [29–65]
Lag time recording–delivery (h)	9 [4–17]	8 [8–11]	12 [6–15]	5 [5–11]

Stage 0: No chorioamnionitis; Stages 1, 2, 3: Diagnosis of chorioamnionitis. Data are represented as median [Interquartile range] for quantitative characteristics and Number (percentage) for qualitative characteristics.

**Table 3:** Comparison between chorioamnionitis and non-chorioamnionitis populations for a selection of features. Only features with significant differences are shown (p-value < 0.05).

Type of features	Feature	Non-chorioamnionitis (Stage 0)	Chorioamnionitis (Stage 1 & 2 & 3)	p-value
cFHR	accelerations number/min	0.07 ± 0.06	0.05 ± 0.05	0.01
	accelerations size/min	0.41 ± 0.45	0.27 ± 0.33	0.005
	LTV	61.34 ± 14.2	55.76 ± 16.41	0.0008
	STV	4.5 ± 1.13	4.04 ± 1.23	0.0006
	EpHV nb/min	0.71 ± 0.19	0.63 ± 0.21	0,002
	EpHV duration/min	0.86 ± 0.18	0.8 ± 0.2	0.007
Time-FHRV	SD	18.66 ± 4.75	17.13 ± 5.97	0.002
	RMSSD	7.43 ± 1.84	6.62 ± 2.09	0.0002
	SDSD	7.43 ± 1.84	6.61 ± 2.09	0.0002
Frequency-FHRV	LF	1.62x10^8^ ± 8.45x10^7^	1.41x10^8^ ± 1.26x10^8^	0.002
	HF	1.14x10^7^ ± 7.21x10^6^	9.42x10^6^ ± 7.3x10^6^	0.002
Nonlinear-FHRV	SD1	5.26 ± 1.3	4.68 ± 1.48	0.0002
	SD2	25.8 ± 6.69	23.76 ± 8.38	0.002
	AC	1.48 ± 0.51	1.31 ± 0.53	0.005
	DC	1.64 ± 0.6	1.42 ± 0.57	0.005

Data are represented as mean ± standard deviation.

### Multiple Factor Analysis

**Fig 3:**
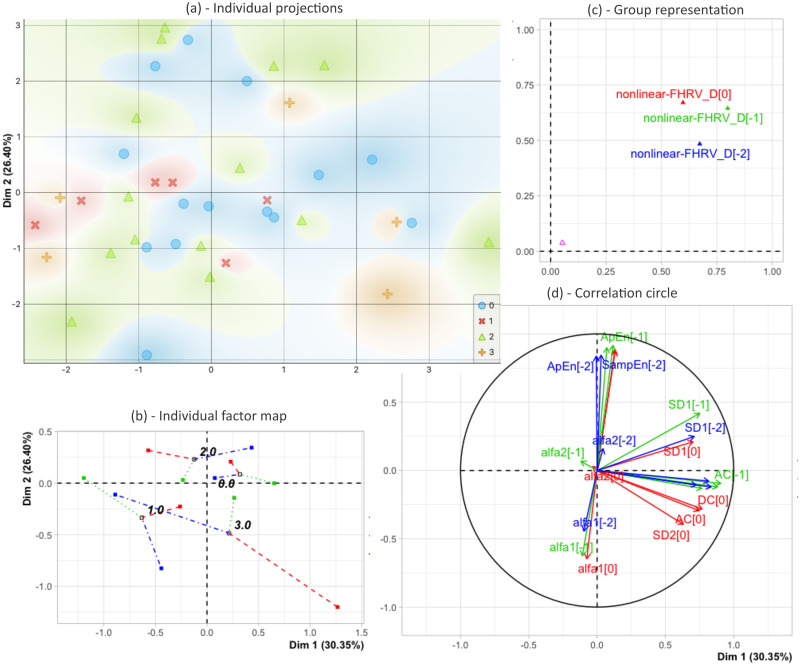
Different results and representations of the multiple factor analysis performed on the nonlinear-FHRV. **(a) Projections of the individuals in the first factorial plane**. The different colors and shapes represent the stages of infection (stage 0: blue circles, stage 1: red cross, stage 2: green triangle, stage 3: orange plus). **(b) Mapping of the partial and global barycenters of the individuals grouped by stage of chorioamnionitis. (c) Groups representation in the first factorial plane. (d) Correlations of the features of the three groups to the first and second dimensions of the MFA**. Red, green and blue in (b), (c) and (d) stand for the groups nonlinear-FHRV for days 0, -1 and -2, respectively. ApEn: Approximate Entropy, SampEn: Sample Entropy, [SD1, SD2]: Poincaré plot indexes, [alfa1, alfa2]: Detrended fluctuation analysis indexes, AC, Acceleration capacity, DC: Deceleration capacity.

As previously explained, the MFA was performed between the last three days before birth on features extracted from cFHR, time-FHRV, frequency-FHRV and nonlinearFHRV. The analyses showed that time-FHRV and nonlinear-FHRV seem to highlight the most distinctions between populations. Consequently, and for the sake of clarity, we have chosen to present in this paragraph an example of the MFA results performed on the HRV features extracted in the non-linear domain only in Fig 3.

The first dimension of the global MFA (λ_1_ = 2.07) explains 30.35% of the total inertia, the second dimension (λ_1_ = 1.8) explains 26.4% of the inertia, representing 56.75% of the information presented in the first factorial plane. The projection of individuals in the first factorial plane (Dim1, Dim2), presented in Fig 3(a), shows that non-chorioamnionitis subjects are more spatially centered than infected subjects. Nevertheless, the distinction between different stages of chorioamnionitis is unclear. The average individual factor map in Fig 3(b) presents the barycenters of each stage of chorioamnionitis from the global MFA, superimposing the three partial MFAs. The global barycenters are thus plotted according to its different variables groups (nonlinear-FHRV D[0], D[-1] and D[-2]). The figure shows that the partial barycenters of stage 0 (non-chorioamnionitis) are more clustered at the global barycenter than those of stages 1, 2 and 3 (chorioamnionitis).

The groups of the MFA are represented on each dimension by the weighted cumulative inertia of the groups’ features in Fig 3(c). The coordinates of the groups show that the first dimension is mainly due to Day[-1] and that Day[0] and Day[-1] both contribute to the second dimension. The proximity of these two groups can be interpreted by the similarity between the variables for days 0 and -1 before birth. The correlation circle in Fig 3(d) shows that the first dimension is correlated with poincaré and acceleration/decelerations features. This dimension is associated to the inter-beats variability. The second dimension is correlated with entropy measures and DFA features, which are related to complexity of RR intervals.

### Distance

**Fig 4:**
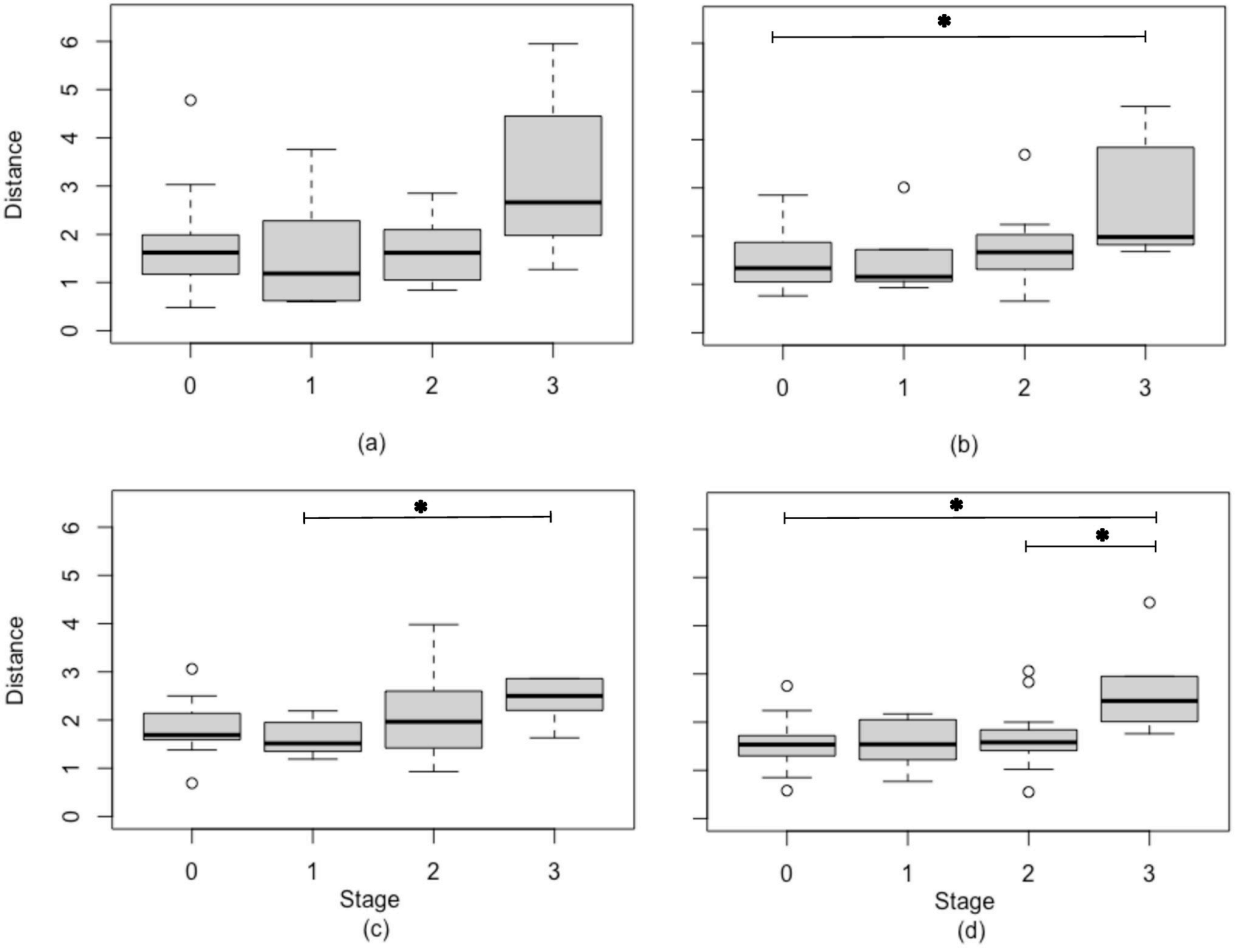
Distances between partial MFA projections of variable groups and global MFA projections for each stage for features from cFHR (a), time-FHRV (b), frequency-FHV (c) and nonlinear-FHRV (d). * is shown when a significant difference (p-value<0.05) was found on statistical tests.

After performing the MFA on all the types of features (cFHR, time-FHRV, frequency-FHRV and nonlinea-FHRV), the 4-dimensional Euclidean distances between the global and partial projections are computed for each individual and represented in boxplots by infection stage (0, 1, 2 and 3) as shown in Fig 4. Distances are compared using the Mann-Whitney test or the Kolmogorov-Smirnov test, depending on whether or not the data follow a normal distribution. Significant differences (p-value < 0.05) are identified on the boxplots by horizontal lines. The boxplots show that distances generally increase with the stage of infection. There is no significant difference between distances in all stages for MFA of cFHR features, while there is a significant difference between stage 3 and stage 0 for the distance computed on MFAs of time-FHRV and nonlinear-FHRV. Other significant differences were observed for MFA of frequency-FHRV (stage 1 vs stage 3) and nonlinear-FHRV (stage2 vs stage3).

### ROC analysis

Table 4 shows the performance of the ROC analysis performed on the distances calculated for each type of features. Firstly, the ROC analysis was performed to distinguish non-chorioamnionitis from all stages of chorioamnionitis (stages 1, 2 and 3). The results show low area under the curve (AUC) for all the types of features, with the highest AUC obtained for time-FHRV (AUC = 62.5% [59.3–65.8] CI). ROC results increased when stage 1 patients were excluded from the ROC analysis, and became higher when only stage 3 patients were considered. Higher AUCs were obtained for nonlinear-FHRV features with AUC = 90.4% [88.2–92.6] CI and for time-FHRV features with AUC = 84.4% [81.8–87.1] CI.

**Table 4:** Areas under the ROC curve of distances calculated on the different types of parameters tested.

	cFHR	time-FHRV	frequency-FHRV	nonlinear-FHRV
**Stage 0 Vs 1&2&3**	40.4 [37–43.7]	62.5 [59.3– 65.8]	52 [48.8–55.2]	60.5 [57.3–63.8]
**Stage 0 Vs 2&3**	48.3 [44.8–51.7]	67.3 [63.9–70.6]	60.3 [57–63.7]	64.6 [61.1–68]
**Stage 0 Vs 3**	72.8 [70.2–75.4]	84.4 [81.8–87.1]	81 [78.2–83.8]	90.4 [88.2–92.6]

## Discussion

This paper presented a prospective study to monitor pregnant women experiencing PPROM between 26 and 34 GW. The aim was to assess the FHR through computerized analysis of FHR and HRV analysis to detect the presence of chorioamnionitis. There are very few studies in the literature that evaluate the FHR to detect chorioamnionitis, yet their findings are ambiguous. To our knowledge, this is the first study in the literature to investigate the fetal heart rate variability. Moreover, the innovation of this study lies in the assessment of the day-to-day relationship using the multiple factor analysis.

The diagnosis of chorioamnionitis remains unclear to this day, leading to confusion and complicating clinical decision-making [30]. As described previously, there is a list of symptoms which help in the clinical diagnosis of chorioamnionitis, but do not have a high predictive value. FHR monitoring is increasingly used in assessing fetal well-being during the intrapartum period, particularly in cases of hypoxia, acidemia and cerebral palsy [9]. However, the relationship between chorioamnionitis and FHR patterns is not yet well established, and previous studies have shown contradictory results. *Salafia et al*. reported that the FHR showed abnormal patterns in cases of chorioamnionitis [10]. In a previous study, the FHR showed increased baseline FHR, reduced variability and loss of acceleration in recordings during labour compared with admissions in chorioamnionitis [15]. Our study compared the features of the last three days before labor between chorioamnionitis and non-chorioamnionitis cases, as in [14]. In accordance with [14, 15], we showed significantly lower accelerations and variability in chorioamnionitis cases, but no significant difference was shown on the baseline FHR. On the other hand, the study by *Kyozuka et al*. showed no association between FHR patterns and chorioamnionitis [30]. Other studies have also reported that FHR abnormalities are not useful in predicting intra-amniotic infection and chorioamnionitis [11–13]. Despite significant differences in certain cFHR features, our study showed that it was difficult to distinguish chorioamnionitis from non-chorioamnionitis cases with low AUC values obtained from the ROC analysis.

In this study, we found it interesting to go beyond standard cFHR features and extract HRV-related features and assess their ability to detect chorioamnionitis. HRV analysis has been shown to have a high predictive value in the diagnosis of several cardiovascular and pulmonary diseases, both in infants and adults [17, 19, 31–33]. The results of our paper suggest that HRV analysis, particularly in the time and nonlinear domains, is useful and provides additional information on fetal health status.

The results of the MFA realized on the FHRV analysis in the nonlinear domain, presented in this paper, showed that there are homogeneous zones for infected and non-infected patients, with no-chorioamnionitis patients mostly projected in the right part of first factorial plane, although overlaps between classes persist. This indicates that no chorioamniotis patients have a better short term HRV adaptation. Similar trends were also observed for features of time-FHRV and frequency-FHRV. This paper also showed that the day-to-day dispersion is higher in subjects with chorioamnionitis than in non-chorioamnionitis subjects. This was highlighted by the 4-dimensional Euclidean distances computed between the global projection and the partial projections of the MFAs for each type of features. This dispersion was more pronounced in subjects with stage 3 choriomanionitis. The ROC analysis performed on the distances, computed from the nonlinear HRV analysis, showed that it was possible to experimentally measure an AUC of 90% when trying to distinguish subjects with stage 3 chorioamnionitis from those without. By grouping stages 2&3 or 1&2&3 in the infected population, distinguishing them from non-chorioamnionitis subjects became more difficult. The AUC decrease respectively around 65% and 60%. The same trends were observed for the others sets of parameters. No improvement were observed when combining all the parameters.

The main limitation of our study is the low number of subjects analyzed and compared in the groups of chorioamnionitis and non-chorioamnionitis. Despite the high number of inclusions made during the clinical study, a very small number of subjects were considered analyzable and retained for data analysis, particularly for subjects who were classified as infected with stage 3 of chorioamnionitis. The study is the initial step for a more generalizable clinical trial, taking into account the study population and the length of the follow-up. The next step would be the inclusion of a greater stage 3 chorioamnionitis population for the confirmation of the interesting observed results compared to the control subjects.

## Conclusion

This study reveals three main findings. Firstly, the assessment of HRV features showed interesting results over cFHR features. Secondly, the assessment of patients’ own dynamics over time seems to be an interesting method for detecting chorioamnionitis. Thirdly, the differentiation is obvious between cases of non-chorioamnionitis and stage 3 chorioamnionitis, whereas it is more subtle for stages 1 and 2 chorioamnionitis.

Future work could include extending the study to other hospital centers, thereby enrolling a larger number of pregnant women with PPROM to improve the generalizability of our findings. It would also be interesting to carry out further analysis to improve the diagnostic performance of features extracted from cFHR, noting that these features can be directly and easily accessible during the FHR recordings.

## Supporting information

S1 Checklist TREND checklist. (PDF)

S1 File Trial study protocol (Original—French version). (PDF)

S2 File Trial study protocol (English version). (PDF)

S1 Table Comparison between chorioamnionitis and non-chorioamnionitis populations for all the extracted features. Features with significant differences are indicated with * and shown in bold.
(PDF)

## References

[1] Schmitz T, Sentilhes L, Lorthe E, Gallot D, Madar H, Doret-Dion M, et al. Preterm premature rupture of the membranes: Guidelines for clinical practice from the French College of Gynaecologists and Obstetricians (CNGOF). European Journal of Obstetrics & Gynecology and Reproductive Biology. 2019;236:1–6. doi: 10.1016/j.ejogrb.2019.02.021 30870741

[2] Baradwan S, AlSghan R, Sabban H, Khadawardi K, Ali ZAM, Felemban LHA, et al. Vaginal probiotics as an adjunct to antibiotic prophylaxis in the management of preterm premature rupture of membranes: A systematic review and meta-analysis of randomized controlled trials. European Journal of Obstetrics & Gynecology and Reproductive Biology. 2023. doi: 10.1016/j.ejogrb.2023.10.011 37862929

[3] Bouvier D, Forest JC, Blanchon L, Bujold E, Pereira B, Bernard N, et al. Risk factors and outcomes of preterm premature rupture of membranes in a cohort of 6968 pregnant women prospectively recruited. Journal of clinical medicine. 2019;8(11):1987. doi: 10.3390/jcm8111987 31731659 PMC6912547

[4] Venkatesh KK, Jackson W, Hughes BL, Laughon MM, Thorp JM, Stamilio DM. Association of chorioamnionitis and its duration with neonatal morbidity and mortality. Journal of Perinatology. 2019;39(5):673–682. doi: 10.1038/s41372-019-0341-x 30723279

[5] Jain VG, Willis KA, Jobe A, Ambalavanan N. Chorioamnionitis and neonatal outcomes. Pediatric research. 2022;91(2):289–296. doi: 10.1038/s41390-021-01633-0 34211129 PMC8720117

[6] Caughey AB, Robinson JN, Norwitz ER. Contemporary diagnosis and management of preterm premature rupture of membranes. Reviews in obstetrics and gynecology. 2008;1(1):11. 18701929 PMC2492588

[7] Du Plessis AH, van Rooyen DR, Jardien-Baboo S, ten Ham-Baloyi W. Screening and diagnosis of women for chorioamnionitis: An integrative literature review. Midwifery. 2022;113:103417. doi: 10.1016/j.midw.2022.103417 35863118

[8] Knupp RJ, Andrews WW, Tita AT. The future of electronic fetal monitoring. Best Practice & Research Clinical Obstetrics & Gynaecology. 2020;67:44–52. doi: 10.1016/j.bpobgyn.2020.02.004 32299728

[9] Evans MI, Britt DW, Evans SM, Devoe LD. Changing perspectives of electronic fetal monitoring. Reproductive Sciences. 2022; p. 1–21. doi: 10.1007/s43032-021-00749-2 34664218 PMC8522858

[10] Salafia CM, Mangam HE, Weigl CA, Foye GJ, Silberman L. Abnormal fetal heart rate patterns and placental inflammation. American journal of obstetrics and gynecology. 1989;160(1):140–147. doi: 10.1016/0002-9378(89)90107-5 2912077

[11] Holcroft CJ, Askin FB, Patra A, Allen MC, Blakemore KJ, Graham EM. Are histopathologic chorioamnionitis and funisitis associated with metabolic acidosis in the preterm fetus? American journal of obstetrics and gynecology. 2004;191(6):2010–2015. doi: 10.1016/j.ajog.2004.05.005 15592284

[12] Sameshima H, Ikenoue T, Ikeda T, Kamitomo M, Ibara S. Association of nonreassuring fetal heart rate patterns and subsequent cerebral palsy in pregnancies with intrauterine bacterial infection. American journal of perinatology. 2005;22(04):181–187. doi: 10.1055/s-2005-867090 15906211

[13] Miyake H, Nakai A, Takeshita T. Fetal heart rate monitoring as a predictor of histopathologic chorioamnionitis in the third trimester. Journal of Nippon Medical School. 2008;75(2):106–110. doi: 10.1272/jnms.75.106 18475031

[14] Vandenbroucke L, Doyen M, Le Lous M, Beuchée A, Loget P, Carrault G, et al. Chorioamnionitis following preterm premature rupture of membranes and fetal heart rate variability. Plos one. 2017;12(9):e0184924. doi: 10.1371/journal.pone.0184924 28945767 PMC5612643

[15] Sukumaran S, Pereira V, Mallur S, Chandraharan E. Cardiotocograph (CTG) changes and maternal and neonatal outcomes in chorioamnionitis and/or funisitis confirmed on histopathology. European Journal of Obstetrics & Gynecology and Reproductive Biology. 2021;260:183–188. doi: 10.1016/j.ejogrb.2021.03.029 33838555

[16] Ponsiglione AM, Cosentino C, Cesarelli G, Amato F, Romano M. A comprehensive review of techniques for processing and analyzing fetal heart rate signals. Sensors. 2021;21(18):6136. doi: 10.3390/s21186136 34577342 PMC8469481

[17] Kabbach EZ, Mazzuco A, Borghi-Silva A, Cabiddu R, Agnoleto AG, Barbosa JF, et al. Increased parasympathetic cardiac modulation in patients with acute exacerbation of COPD: how should we interpret it? International Journal of Chronic Obstructive Pulmonary Disease. 2017; p. 2221–2230. doi: 10.2147/COPD.S134498 28814850 PMC5546179

[18] Sullivan BA, Fairchild KD. Vital signs as physiomarkers of neonatal sepsis. Pediatric research. 2022;91(2):273–282. doi: 10.1038/s41390-021-01709-x 34493832 PMC13064443

[19] Koppens HJ, Onland W, Visser DH, Denswil NP, van Kaam AH, Lutterman CA. Heart Rate Characteristics Monitoring for Late-Onset Sepsis in Preterm Infants: A Systematic Review. Neonatology. 2023;120(5):548–557. doi: 10.1159/000531118 37379804 PMC10614451

[20] Des Jarlais DC, Lyles C, Crepaz N, Group T. Improving the reporting quality of nonrandomized evaluations of behavioral and public health interventions: the TREND statement. American journal of public health. 2004;94(3):361–366. doi: 10.2105/ajph.94.3.361 14998794 PMC1448256

[21] Sentilhes L, Sénat MV, Ancel PY, Azria E, Benoist G, Blanc J, et al. Prevention of spontaneous preterm birth: Guidelines for clinical practice from the French College of Gynaecologists and Obstetricians (CNGOF). European Journal of Obstetrics & Gynecology and Reproductive Biology. 2017;210:217–224. doi: 10.1016/j.ejogrb.2016.12.035 28068594

[22] Jones GD, Cooke WR, Vatish M, Redman CW. Computerized analysis of antepartum cardiotocography: a review. Maternal-Fetal Medicine. 2022;4(2):130–140. doi: 10.1097/FM9.0000000000000141 PMC12094408 40406442

[23] Shaffer F, Ginsberg JP. An overview of heart rate variability metrics and norms. Frontiers in public health. 2017; p. 258. doi: 10.3389/fpubh.2017.00258 29034226 PMC5624990

[24] Lomb NR. Least-squares frequency analysis of unequally spaced data. Astrophysics and space science. 1976;39:447–462. doi: 10.1007/BF00648343

[25] Bauer A, Kantelhardt JW, Barthel P, Schneider R, Mäkikallio T, Ulm K, et al. Deceleration capacity of heart rate as a predictor of mortality after myocardial infarction: cohort study. The lancet. 2006;367(9523):1674–1681. doi: 10.1016/S0140-6736(06)68735-7 16714188

[26] Pagès J. Multiple factor analysis by example using R. CRC Press; 2014.

[27] Abdi H, Williams LJ. Principal component analysis. Wiley interdisciplinary reviews: computational statistics. 2010;2(4):433–459. doi: 10.1002/wics.101

[28] Abdi H, Williams LJ, Valentin D. Multiple factor analysis: principal component analysis for multitable and multiblock data sets. Wiley Interdisciplinary reviews: computational statistics. 2013;5(2):149–179. doi: 10.1002/wics.1246

[29] Lê S, Josse J, Husson F. FactoMineR: an R package for multivariate analysis. Journal of statistical software. 2008;25:1–18.

[30] Kyozuka H, Yasuda S, Hiraiwa T, Ishibashi M, Kato K, Fujimori K. Histological chorioamnionitis as a risk factor for preterm birth without disturbing fetal heart rate: a case-control study. The Tohoku Journal of Experimental Medicine. 2017;243(4):289–295. doi: 10.1620/tjem.243.289 29249732

[31] Williams DP, Koenig J, Carnevali L, Sgoifo A, Jarczok MN, Sternberg EM, et al. Heart rate variability and inflammation: a meta-analysis of human studies. Brain, behavior, and immunity. 2019;80:219–226. doi: 10.1016/j.bbi.2019.03.009 30872091

[32] León C, Carrault G, Pladys P, Beuchée A. Early detection of late onset sepsis in premature infants using visibility graph analysis of heart rate variability. IEEE Journal of Biomedical and Health Informatics. 2020;25(4):1006–1017. 10.1109/JBHI.2020.3021662 32881699

[33] Chiera M, Cerritelli F, Casini A, Barsotti N, Boschiero D, Cavigioli F, et al. Heart rate variability in the perinatal period: a critical and conceptual review. Frontiers in Neuroscience. 2020;14:561186. doi: 10.3389/fnins.2020.561186 33071738 PMC7544983

