# Supplementary material for: Day-to-day dynamics of fetal heart rate variability to detect chorioamnionitis in preterm premature rupture of membranes
Source: PLoS One. 2025 Jan 2;20(1):e0305875. doi: 10.1371/journal.pone.0305875 (PMC11695014; doi:10.1371/journal.pone.0305875)
Supplement: S1 File — (PDF) [file pone.0305875.s002.pdf]

Analyse informatisée de la variabilité du rythme cardiaque fœtal pour le dépistage précoce de la chorioamniotite dans les ruptures prématurées des membranes avant terme

Responsable de l'étude : CHU de Rennes, Hôpital de Pontchaillou, 2 rue Henri le Guilloux, 35033 Rennes cedex 9

Investigateur principal : Dr Linda Lassel
Service de Gynécologie-Obstétrique et Médecine de la Reproduction, CHU de Rennes, Hôpital Sud, 16 boulevard de Bulgarie, 35203 Rennes cedex 2
Unité SEPIA - Laboratoire Traitement du Signal et de l'Image (LTSI, UMR INSERM 1099), Université de Rennes 1, Campus de Beaulieu, Bâtiment 22, 35042 Rennes cedex
Tél : 06.34.18.25.88 – Email : linda.lassel@chu-rennes.fr

Méthodologiste :
Dr Bruno Laviolle, Centre d'Investigation Clinique, Inserm 1414, Unité de Pharmacologie Clinique, Hôpital de Pontchaillou, 2 rue Henri le Guilloux, 35033 Rennes cedex 9. Tél : 02.99.28.96.68 – Email : bruno.laviolle@chu-rennes.fr

Monitoring : Direction de la Recherche Clinique, Hôpital de Pontchaillou, 2 rue Henri le Guilloux, 35033 Rennes cedex 9. Tél : 02.99.28.25.55 – Email : drc@chu-rennes.fr

Historique des versions

Version soumise au CER : 1.0 du 14 octobre 2014

Version acceptée au CER: 2.0 du 17 novembre 2014

Version transmise pour information : 4.0 du 8 décembre 2014

Version transmise pour modification n°2 : 5.4 du 16 janvier 2017

Version modifiée acceptée au CER : 6.0 du 21/01/2017

Version transmise pour modification n°3 : 7.0 du 23/02/2018

Version modifiée acceptée au CER : 8.0 du 20/04/2018

Version transmise pour modification n°3 : 7.0 du 23/02/2018

Version modifiée acceptée au CER : 8.0 du 20/04/2018

Version transmise pour modification n°4 : 9.0 du 31/08/2018

Version modifiée acceptée au CER : 10.0 du 25/09/2018

Version transmise pour modification n°5 : 11.0 du 16/11/2020

Version modifiée acceptée au CER : 12.0 du 17/11/2020

Version modifiée transmise pour information au CER n°6 : 14.0 du 08/11/2021

RESPONSABLE DE L'ETUDE :	CHU DE RENNES
**PROTOCOLE D'ESSAI CLINIQUE**<br>**AiRPM**	
**CODE ESSAI**	35RC14_9772
**TITRE COMPLET**	Analyse informatisée de la variabilité du rythme cardiaque fœtal pour le dépistage précoce de la chorioamniotite dans les ruptures prématurées des membranes avant terme
**INDICATION(S) (CIBLE)**	Rupture prématurée des membranes avant terme
**INVESTIGATEUR PRINCIPAL**	Dr Linda Lassel<br>Service de Gynécologie-Obstétrique et Médecine de la Reproduction<br>CHU de Rennes<br>Hôpital Sud<br>16 Boulevard de Bulgarie<br>35203 Rennes cedex 2
**N° DE VERSION DU PROTOCOLE**	Version 14.0
**DATE DU PROTOCOLE**	8 novembre 2021
**CER**	*Date d'avis : 10 novembre 2014*<br>*N° d'avis : 14.76*
**CNIL**	*Date de déclaration : 17octobre 2014*<br>*N° de déclaration : 1802940 v 0*<br>*Engagement de conformité MR003 : 7 novembre 2016*
**CE DOCUMENT CONFIDENTIEL EST LA PROPRIETE DU CHU DE RENNES**<br>**AUCUNE INFORMATION NON PUBLIEE FIGURANT DANS CE DOCUMENT NE PEUT ETRE DIVULGUEE SANS AUTORISATION ECRITE PREALABLE DU CHU DE RENNES**	

SOMMAIRE

1. INFORMATIONS GENERALES 14
1.1. Titre 14
1.2. Responsable 14
1.3. Coordination et suivi de l'étude 14
1.4. Investigateur principal 14
1.5. Investigateur(s) associé(s) 15
1.6. Scientifiques associés 15
1.7. Méthodologiste 15
1.8. Appuis organisationnel et scientifique 15
2. JUSTIFICATION DE L'ETUDE 15
3. OBJECTIFS 19
3.1. Objectif principal 19
3.2. Objectif(s) secondaire(s) 19
4. DEFINITION DES SUJETS ELIGIBLES 19
4.1. Critères d'inclusion 19
4.2. Critères de non inclusion 19
4.3. Critère d'exclusion pour l'objectif principal 20
5. CRITERES DE JUGEMENT 20
5.1. Critère principal 20
5.2. Critère(s) secondaire(s) 20
6. IDENTIFICATION DU DISPOSITIF MÉDICAL 20
7. CONCEPTION DE LA RECHERCHE 21
7.1. Méthodologie de la recherche 21
7.2. Déroulement de la recherche 21
8. NOMBRE DE SUJETS NECESSAIRE 24
9. ANALYSE STATISTIQUE 24
10. FAISABILITE DU PROJET 24
11. ASPECTS LOGISTIQUES, LEGAUX ET GENERAUX 25
11.1. Calendrier prévisionnel 25
11.2. Comité d'éthique 25
11.3. CNIL 25
11.4. Modifications substantielles 25
11.5. Information et non opposition 25
11.6. Confidentialité des données 25
11.7. Contrôle et assurance de la qualité 25
11.8. Recueil des données 26
11.9. Archivage 26
11.10. Assurance 26

11.11. Règles relatives à la publication 26
12. BIBLIOGRAPHIE **27**
13. LISTE DES ANNEXES **28**

PAGE DE SIGNATURES

INVESTIGATEUR PRINCIPAL

J'ai lu l'ensemble des pages du protocole de l'essai clinique dont le CHU de Rennes est le responsable. Je confirme qu'il contient toutes les informations nécessaires à la conduite de l'essai. Je m'engage à réaliser l'essai en respectant le protocole et les termes et conditions qui y sont définis. Je m'engage à réaliser l'essai en respectant les principes de la "Déclaration d'Helsinki".

Je m'engage également à ce que les investigateurs et les autres membres qualifiés de mon équipe aient accès aux copies de ce protocole et des documents relatifs à la conduite de l'essai pour leur permettre de travailler dans le respect des dispositions figurant dans ces documents.

NOM DE L'INVESTIGATEUR PRINCIPAL : **Docteur Linda LASSEL**

Signature : ………………………………Date : …………………………………….

RESPONSABLE DE L'ETUDE

Responsable de l'étude : CHU de RENNES

NOM : **Nicolas MEVEL– Directeur de la Recherche**

Signature : ……………………………   Date : ……………………………

RESUME

TITRE	Analyse informatisée de la variabilité du rythme cardiaque fœtal pour le dépistage précoce de la chorioamniotite dans les ruptures prématurées des membranes avant terme
RESPONSABLE DE L'ETUDE	CHU de Rennes
INVESTIGATEUR PRINCIPAL	Dr Linda Lassel<br>Service de Gynécologie-Obstétrique et Médecine de la Reproduction<br>CHU de Rennes<br>Hôpital Sud<br>16 Boulevard de Bulgarie<br>35203 Rennes cedex 2
VERSION DU PROTOCOLE	14.0 du 08 novembre 2021
JUSTIFICATION/CONTEXTE	La chorioamniotite correspond à une inflammation ou une infection, le plus souvent d'origine bactérienne, de la cavité ovulaire. Elle apparaît secondairement à la rupture prématurée des membranes (RPM) dans la majorité des cas. La RPM est définie par l'ouverture de la poche des eaux douze heures ou plus avant la mise en travail.<br>La RPM avant terme (< 37 semaines d'aménorrhées) est un problème majeur de santé publique. Elle représente 2 à 3% des grossesses et environ un tiers des accouchements prématurés soit plus de 20 000 naissances par an en France. Au-delà de la prématurité, le pronostic néonatal en cas de RPM avant terme est directement influencé par la présence d'une chorioamniotite, estimée entre 15 et 36% des cas selon les auteurs.<br>En effet, la chorioamniotite est responsable d'une aggravation de la morbi/mortalité néonatale en cas d'accouchement prématuré. Les phénomènes inflammatoires/infectieux induits par la chorioamniotite augmentent la fréquence des détresses respiratoires, des pathologies pulmonaires infectieuses (pneumopathies, alvéolites), des entérocolites nécrosantes, des leucomalacies périventriculaires ainsi que des hémorragies intracrâniennes et ce, indépendamment de la prématurité. La chorioamniotite majore aussi le risque de survenue de maladies de la substance blanche à l'origine d'infirmités motrices cérébrales. Cette relation a été largement documentée *in vitro*, par des études expérimentales chez l'animal ainsi que lors d'études observationnelles chez l'humain.<br>On retrouve principalement deux définitions de la chorioamniotite dans la littérature :<br>- la chorioamniotite histologique (définition la plus consensuelle et retenue dans le cadre de ce projet de recherche) correspond à une atteinte inflammatoire du placenta (inflammation maternelle) et

	éventuellement du cordon ombilical (réaction inflammatoire fœtale) lors de l'examen anatomopathologique du placenta.<br>- la chorioamniotite clinique, caractérisée par la survenue de signes cliniques évocateurs d'une inflammation/infection de la cavité utérine et/ou du fœtus.<br>Ainsi, les stratégies utilisées pour diminuer ce risque de chorioamniotite en cas de RPM avant terme sont, d'une part la prescription systématique d'antibiotiques et, d'autre part, une surveillance clinique et biologique rapprochée afin de détecter sa survenue de la manière la plus précoce possible. Le cas échéant, la naissance de l'enfant, même prématurée, est provoquée afin d'éviter d'aggraver le pronostic néonatal en surajoutant les complications de la chorioamniotite à celles de la prématurité.<br>Néanmoins, il n'existe actuellement pas de marqueur spécifique permettant la détection précoce de la chorioamniotite :<br>- les signes cliniques de la chorioamniotite sont inconstants, variables, non spécifiques et interviennent le plus souvent à un stade avancé de l'infection (contractions utérines, hyperthermie, tachycardie maternelle et/ou fœtale, utérus douloureux à la palpation, liquide amniotique fétide).<br>- la valeur prédictive positive des marqueurs biologiques utilisés en pratique clinique courante est très faible, en particulier pour la CRP et l'hyperleucocytose qui sont les plus utilisées en France. Le dosage de certaines cytokines semble prometteur mais n'est encore qu'expérimental et ne fait pas l'objet de larges séries. De plus, bien que la sensibilité de ces cytokines soit intéressante, aucune d'entre elles n'est spécifique de l'infection fœtale ou intra-amniotique.<br>Ainsi, il manque dans la pratique clinique courante un ou des marqueurs spécifique(s), non invasif(s) et facilement accessible(s) qui permettraient une détection précoce de la chorioamniotite en cas de RPM avant terme.<br>Notre étude monocentrique réalisée au CHU de Rennes (annexe 2) sur une cohorte de 23 patientes avec une RPM avant terme a permis de montrer pour la première fois que l'analyse de la variabilité du rythme cardiaque fœtal (RCF) est une voie de recherche prometteuse pour le dépistage de la chorioamniotite. En effet, on observe une modification de paramètres de la variabilité cardiaque fœtale en cas de chorioamniotite histologique dans le contexte d'une RPM avant terme. Parmi ces modifications, on retrouve une augmentation de la fréquence cardiaque de base (p=0,02), une augmentation des épisodes de basse variation (p=0,04), une diminution de la variation à court terme (p=0,003) et des épisodes de haute variation (p < 0,001) dans les derniers enregistrements réalisés avant la naissance (spontanée ou provoquée) en cas de chorioamniotite. L'indice des

	épisodes de haute variation (rapport entre la moyenne du nombre d'épisodes de haute variation des deux derniers enregistrements et les quatre précédents) semble un outil prometteur pour le diagnostic précoce de la chorioamniotite dans les RPM avant terme (sensibilité 90%, spécificité 84,6%, valeur prédictive positive 71,5%, valeur prédictive négative 95,2%, aire sous la courbe ROC = 0,88, intervalle de confiance à 95% 0,73-100). Ces données vont dans le sens de celles observées en cas d'infection néonatale et sont cohérentes avec les mécanismes physiopathologiques sous-jacents (perte de la variabilité, diminution de l'adaptabilité fœtale en réaction au stimulus infectieux/inflammatoire placentaire).<br>En conclusion, le dépistage précoce de la chorioamniotite dans les RPM avant terme est un enjeu majeur de la recherche en obstétrique et il manque encore aujourd'hui un outil diagnostique efficace. Si les résultats de notre étude pilote étaient confirmés, l'intégration de ces marqueurs de la variabilité cardiaque dans un monitoring temps-réel répondrait à cet objectif de manière simple, non invasive et reproductible.
OBJECTIF PRINCIPAL	L'objectif principal est de mesurer la valeur diagnostique de l'indice des épisodes de haute variation du rythme cardiaque fœtal (rapport entre la moyenne du nombre d'épisodes de haute variation des deux derniers enregistrements et les quatre précédents) pour la détection de la chorioamniotite prouvée histologiquement dans les RPM avant terme.
OBJECTIFS SECONDAIRES	Les objectifs secondaires sont :<br>- caractériser l'évolution des paramètres de la variabilité cardiaque fœtale et néonatale (analyse linéaire et non linéaire, cf annexe 1) sur une population de RPM avant terme avec et sans chorioamniotite histologique.<br>- tester l'hypothèse que les autres paramètres d'analyse de la variabilité cardiaque fœtale peuvent constituer, seuls ou en association notamment avec l'indice des épisodes de haute variation un indicateur uni ou multivarié de la chorioamniotite histologique dans les RPM avant terme.
CRITERE DE JUGEMENT PRINCIPAL	Le critère de jugement principal est l'indicateur d'efficacité (aire sous courbe) ROC, sensibilité, spécificité, valeur prédictive positive, valeur prédictive négative) de l'indice des épisodes de haute variation pour la détection de la chorioamniotite histologique dans les RPM avant terme.
CRITERES DE JUGEMENT SECONDAIRES	- Les performances de l'approche seront aussi testées par les changements observés dans l'évolution des paramètres de la variabilité cardiaque fœtale et néonatale (analyse linéaire et non linéaire, cf annexe 1). L'expression quantitative (analyse statistique) et modélisée (analyse graphique et statistique) de l'évolution de ces marqueurs permettra de

	définir leur valeur diagnostique pour le diagnostic de chorioamniotite histologique et/ou d'infection materno-fœtale dans les RPM avant terme.<br>- Un indicateur d'efficacité (aire sous courbe ROC, sensibilité, spécificité, valeur prédictive positive, valeur prédictive négative) sera aussi utilisé pour évaluer la valeur diagnostique des autres paramètres d'analyse de la variabilité cardiaque retenus sous la forme d'indicateur soit uni ou multivarié pour la détection de la chorioamniotite histologique dans les RPM avant terme.
METHODOLOGIE / SCHEMA DE L'ETUDE	Etude contrôlée cas-témoins monocentrique d'observation avec analyse en insu du personnel soignant. L'inclusion sera prospective : toute patiente enceinte d'une grossesse singleton hospitalisée pour RPM survenue entre 26 et 34 SA sera potentiellement incluse dans l'étude.<br>Le recueil doppler/ECG du signal cardiaque sera prospectif, extrait des enregistrements réalisés dans le cadre de la surveillance habituelle du bien être fœtal et néonatal. L'analyse des paramètres de variabilité cardiaque (indice des épisodes de haute variation, analyse linéaire et non linéaire) sera réalisée à l'aide du logiciel Matlab® en insu du personnel soignant. Les algorithmes de calcul de ces paramètres ont déjà été développés par le Laboratoire Traitement du Signal et de l'Image (LTSI).
CRITERES D'INCLUSION	- patiente majeure<br>- grossesse singleton<br>- RPM survenue entre 26 et 34 SA, authentifiée à l'examen clinique et si doute, confirmée par un test diagnostique vaginal détectant l'IGFBP-1.<br>- patiente ayant reçu l'information sur le protocole et n'ayant pas manifesté son opposition à participer

CRITERES DE NON INCLUSION	- grossesse multiple<br>- hypotrophie néonatale (poids de naissance <10ème percentile AUDIPOG)<br>- tabagisme maternel actif<br>- diabète gestationnel ou préexistant à la grossesse<br>- pathologie maternelle :<br>o cardiopathie congénitale ou acquise<br>o embolie pulmonaire en cours de traitement<br>o hypertension artérielle pulmonaire<br>o insuffisance rénale chronique modérée à sévère<br>o bronchopneumopathie chronique obstructive<br>o maladie auto-immune (lupus érythémateux disséminé, sclérose en plaques, syndrome de Gougerot-Sjögren)<br>- malformation fœtale cardiaque, neurologique ou génétique avérée
CRITERE D'EXCLUSION POUR L'OBJECTIF PRINCIPAL	Accouchement dans les 48h suivant la survenue de la RPM (situation clinique ne permettant pas le calcul de l'indice des épisodes de haute variation ni de modéliser l'évolution des paramètres d'analyse de la VRC)
STRATEGIE/PRODUIT/DISPOSITIF MEDICAL	Le recueil doppler du signal cardiaque fœtal sera effectué à l'aide d'un moniteur cardiotocographique F3 Fetal Monitor (EDAN Instruments, Inc ; figure 1) qui dispose des caractéristiques techniques communes à tous les cardiotocographes concurrents. Ce dispositif a l'avantage d'être muni d'une mémoire interne de 60 heures permettant de stocker les enregistrements effectués sous forme de fichiers TRC (Trace file) indépendants, transférables sur ordinateur via un port USB. Ces fichiers TRC permettent d'obtenir sous forme numérique le relevé des battements cardiaques fœtaux rééchantillonnés à 4 Hz et de procéder ainsi au calcul des paramètres de variabilité cardiaque sous Matlab®.<br>Le recueil du signal cardiaque néonatal par ECG sera réalisé 48h après la naissance, puis 1 fois par semaine.
NOMBRE DE PATIENTES	120 patientes à inclure dont 60 patientes analysables pour l'objectif principal (calcul basé sur les résultats de l'étude pilote réalisée au CHU de Rennes)
NOMBRE THEORIQUE DE CENTRES	4 centres d'HUGOPEREN (Angers, Nantes, Rennes et Poitiers)
DUREE DE LA RECHERCHE	Durée de la période d'inclusion : 39 mois<br>Durée maximale de la participation pour chaque couple patiente/nouveau-né : en moyenne 4 semaines de suivi pour la mère, et jusqu'à 15 semaines pour le nouveau-né, soit environ 19 semaines<br>Durée maximale totale de l'étude : 50 mois

RETOMBEES ATTENDUES	Ce projet doit positionner le CHU de Rennes comme le leader français mais aussi européen dans la recherche technologique et clinique pour la prise en charge en obstétrique.<br>• Perspectives médicales : à terme, fournir aux cliniciens un outil d'aide à la décision en intégrant les indicateurs proposés dans un système de monitoring temps-réel qui permettra un diagnostic précoce de la chorioamniotite dans les RPM avant terme de façon rapide, non invasive, reproductible et plus efficace que les moyens existants. L'amélioration de la performance de ce diagnostic permettrait une diminution importante de la morbi/mortalité néonatale en évitant de surajouter les complications de la chorioamniotite à celles de la prématurité en cas de RPM avant terme.<br>• Perspectives fondamentales : création de nouveaux outils en traitement du signal, compréhension de la maturation des rythmes au cours du développement.<br>• Perspectives industrielles : création des systèmes d'aide au diagnostic médical de la chorioamniotite et de l'infection maternofœtale avec dépôt de brevet, nouvelles méthodes de monitoring et d'explorations fonctionnelles multiparamétriques.<br>• Perspectives en recherche : évaluation finale d'ici 2 à 3 ans dans un projet de recherche clinique multicentrique randomisée (PHRC national) en intention de traiter ; création d'un réseau d'étude sur la variabilité des contrôles et régulations du rythme cardiaque au cours du développement.

LISTE DES ABREVIATIONS

ANOVA	ANalysis Of Variance
ASCENT	Anonymised System for Clinical Experimentation
ApEn	Approximate entropy
ARC	Attaché de Recherche Clinique
AUDIPOG	Association des Utilisateurs de Dossiers Informatisés en Pédiatrie, Obstétrique et Gynécologie
CER	Comité d'Ethique de Rennes
CHU	Centre Hospitalier Universitaire
CIC-IT	Centre d'Investigation Clinique - Innovation Technologique
CNIL	Commission nationale de l'informatique et des libertés
CRF	Case Report Form
CRP	C-reactive protein
DFA	Detrended Fluctuation Analysis
ECG	Electrocardiographie
ETF	Echographie Transfontanellaire
ERCF	Enregistrement du rythme cardiaque fœtal
GIRCI	Groupement Interrégional de Recherche Clinique et d'Innovation
HF	High frequency
HTAP	Hypertension Artérielle Pulmonaire
IGFBP-1	Insulin-like growth factor-binding protein 1
INSERM	Institut national de la santé et de la recherche médicale
IRM	Imagérie par Résonnance Magnétique
LF	Low frequency
LTSI	Laboratoire Traitement du Signal et de l'Image
NICE	National Institute for Health and Care Excellence
NN	Normal to normal
PHRC	Programme Hospitalier de Recherche Clinique
PNN	Polynucléaires neutrophiles
RCF	Rythme cardiaque foetal
rMSSD	root mean square of successive differences
ROC	Receiver operating characteristic
RPM	Rupture prématurée des membranes
SA	Semaine d'aménorrhée
SampEn	Sample entropy
SD	Standard deviation
SDNN	Standard deviation of NN intervals
TRC	Trace file
ULF	Ultra low frequency
UMR	Unité Mixte de Recherche

USB	Universal Serial Bus
VCT	Variation à court terme
VLF	Very low frequency
VRC	Variabilité du rythme cardiaque

1. INFORMATIONS GENERALES

1.1. Titre

Analyse informatisée de la variabilité du rythme cardiaque fœtal pour le dépistage précoce de la chorioamniotite dans les ruptures prématurées des membranes avant terme

1.2. Responsable

Identité

CHU de Rennes
2, rue Henri le Guilloux
35033 Rennes Cedex 9

Signature du protocole au nom du responsable

Monsieur Pascal GAUDRON – Directeur de la Recherche
CHU de Rennes – Hôpital de Pontchaillou
2, rue Henri le Guilloux
35033 Rennes Cedex 9

Responsable de la recherche

Monsieur Pascal GAUDRON– Directeur de la Recherche
CHU de Rennes – Hôpital de Pontchaillou
2, rue Henri le Guilloux
35033 Rennes Cedex 9

1.3. Coordination et suivi de l'étude

Direction de la Recherche
CHU de Rennes – Hôpital de Pontchaillou
2, rue Henri le Guilloux
35033 Rennes Cedex 9

1.4. Investigateur principal

Dr Linda Lassel
Service de Gynécologie-Obstétrique
CHU de Rennes
Hôpital Sud
16 Boulevard de Bulgarie

35203 Rennes cedex 2

1.5. Investigateur(s) associé(s)

Dr Christelle Mainguy

Service de Gynécologie-Obstétrique

CHU de Rennes

Hôpital Sud

16 Boulevard de Bulgarie

35203 Rennes cedex 2

Dr Pierre-Emmanuel Bouet

Service de Gynécologie-Obstétrique

CHU d'Angers

4 rue Larrey

49933 Angers Cedex 9

Dr Vincent Dochez

Service de Gynécologie-Obstétrique

CHU de Nantes

38 Boulevard Jean Monnet

44093 Nantes Cedex 1

Dr Bertrand GACHON

Service de Gynécologie-Obstétrique

CHU de Poitiers

2 Rue de la Milétrie

86021 Poitiers Cedex

1.6. Scientifiques associés

Pr Guy Carrault

Laboratoire Traitement du Signal et de l'Image (INSERM UMR1099) – Equipe SEPIA

Campus de Beaulieu, Université de Rennes 1

35042 Rennes Cedex

Pr Patrick Pladys

Laboratoire Traitement du Signal et de l'Image (INSERM UMR1099) – Equipe SEPIA

Campus de Beaulieu, Université de Rennes 1

35042 Rennes Cedex

Dr David Riochet
Coordination du réseau de recherche pédiatrique du Grand Ouest HUGOPEREN
HME
CHU de Nantes
44093 Nantes cedex 01
Email : david.riochet@univ-nantes.fr

1.7. Méthodologiste

Dr Bruno Laviolle, Centre d'Investigation Clinique, Inserm 1414, Unité de Pharmacologie Clinique, Hôpital de Pontchaillou, 2 rue Henri le Guilloux, 35033 Rennes cedex 9. Tél : 02.99.28.96.68 – Email : bruno.laviolle@chu-rennes.fr

2. JUSTIFICATION DE L'ETUDE

Rupture prématurée des membranes avant terme et dépistage précoce de la chorioamniotite

La chorioamniotite correspond à une inflammation ou une infection, le plus souvent d'origine bactérienne, de la cavité ovulaire. Elle apparaît secondairement à la rupture prématurée des membranes (RPM) dans la majorité des cas (1).

La RPM est définie par l'ouverture de la poche des eaux douze heures ou plus avant la mise en travail. Le diagnostic est avant tout clinique par la visualisation à l'examen clinique d'un écoulement de liquide amniotique. En cas de doute, le diagnostic est facilité par la réalisation de tests dont le plus sensible et spécifique est, à ce jour, la détection de l'IGFBP-1 dans les pertes vaginales (2).

La RPM avant terme (< 37 semaines d'aménorrhée) est un problème majeur de santé publique. Elle représente 2 à 3% des grossesses et environ un tiers des accouchements prématurés soit plus de 20 000 naissances par an en France (3-5). Au delà de la prématurité, le pronostic néonatal en cas de RPM avant terme est directement influencé par la présence d'une chorioamniotite, estimée entre 15 et 36% des cas selon les auteurs (6).

En effet, la chorioamniotite est responsable d'une aggravation de la morbi/mortalité néonatale en cas d'accouchement prématuré. Les phénomènes inflammatoires/infectieux induits par la chorioamniotite augmentent la fréquence des détresses respiratoires, des pathologies pulmonaires infectieuses (pneumopathies, alvéolites), des entérocolites nécrosantes, des leucomalacies périventriculaires ainsi que des hémorragies intracrâniennes et ce, indépendamment de la prématurité (6-8). La chorioamniotite majore aussi le risque de survenue de maladies de la substance blanche à l'origine d'infirmités motrices cérébrales. Cette relation a été largement documentée *in vitro*, par des études expérimentales chez l'animal ainsi que lors d'études observationnelles chez l'humain (9-13).

On retrouve principalement deux définitions de la chorioamniotite dans la littérature :

- la plus consensuelle est histologique, par la mise en évidence à l'examen anatomopathologique du placenta d'une inflammation chorionique (infiltrat de polynucléaires neutrophiles dans la plaque choriale) (1). L'inflammation associée des vaisseaux ombilicaux (funiculite), engendrée par la réponse inflammatoire fœtale, représente un facteur de gravité (14). L'inconvénient de cette définition dans la pratique clinique courante est le caractère rétrospectif du diagnostic.
- la chorioamniotite clinique, caractérisée par la survenue de signes cliniques évocateurs d'une

inflammation/infection de la cavité utérine et/ou du fœtus.

Si après 34 semaines d'aménorrhée (SA), la conduite à tenir n'est pas consensuelle entre l'expectative avec surveillance ou la décision de la naissance, en revanche, avant 34 SA, la prise en charge des RPM avant terme consiste en une attitude d'expectative avec surveillance maternofœtale rapprochée dans une unité de grossesses à haut risque de niveau adapté à l'âge gestationnel (15). La mise en évidence de signes faisant suspecter l'installation d'une chorioamniotite conduit, à l'inverse, à l'arrêt de la grossesse et à une naissance urgente (16).

Ainsi, les stratégies utilisées pour diminuer ce risque de chorioamniotite en cas de RPM avant terme sont, d'une part la prescription systématique d'antibiotiques et, d'autre part, une surveillance clinique et biologique rapprochée afin de détecter sa survenue de la manière la plus précoce possible.

Néanmoins, il n'existe actuellement pas de marqueur spécifique permettant la détection précoce de la chorioamniotite :

- les signes cliniques de la chorioamniotite sont inconstants, variables, non spécifiques et interviennent le plus souvent à un stade avancé de l'infection (contractions utérines, hyperthermie, tachycardie maternelle et/ou fœtale, utérus douloureux à la palpation, liquide amniotique fétide) (17).
- la valeur prédictive positive des marqueurs biologiques utilisés en pratique clinique courante est très faible, en particulier pour la CRP et l'hyperleucocytose qui sont les plus utilisées en France. Le dosage de certaines cytokines semble prometteur mais n'est encore qu'expérimental et ne fait pas l'objet de larges séries. De plus, bien que la sensibilité de ces cytokines soit intéressante, aucune d'entre elles n'est spécifique de l'infection fœtale ou intra-amniotique (18).

Des travaux réalisés au CHU de Rennes (annexe 2) ont montré pour la première fois que l'analyse de la variabilité du rythme cardiaque fœtal (RCF) est une voie de recherche prometteuse dans cette indication.

L'étude de la variabilité cardiaque fœtale et néonatale : une nouvelle voie de recherche pour le dépistage précoce de la chorioamniotite et de l'infection materno-foetale dans les RPM avant terme

L'étude de la variabilité du rythme cardiaque (VRC) permet d'approcher de façon non invasive l'activité du système nerveux autonomique. La VRC est généralement calculée par analyse des séries chronologiques battement par battement des intervalles RR de l'ECG.

Diverses méthodes d'analyse de la variabilité du rythme cardiaque ont été proposées et de nombreux paramètres en sont extraits :

- l'analyse linéaire qui se décompose en :
  - analyse temporelle avec le calcul des paramètres SD, rMSSD
  - analyse fréquentielle où l'on mesure des puissances dans des bandes pré-determinées appelées LF, HF, VLF
- l'analyse non linéaire où différents indices sont estimés par exemple à partir du Diagramme de Poincaré (SD1, SD2) ou directement à partir des séries temporelles telles que l'entropie ApEn, SampEn, DFA-α1 (cf Annexe 1)

Les applications diagnostiques et pronostiques de l'étude de la VRC sont nombreuses, que ce soit chez l'adulte (mort subite, athérosclérose, insuffisance cardiaque, syndrome d'apnées du sommeil, neuropathie diabétique) (19) ou en néonatologie : mort subite du nourrisson (20), infection néonatale tardive. Il est en effet possible d'identifier chez le nouveau-né prématuré la survenue d'infections néonatales tardives (supérieures à 3 jours de vie) en

analysant la complexité du rythme cardiaque et en caractérisant les ralentissements cardiaques. Ainsi, le sepsis chez les nouveaux-nés prématurés est associé à une baisse de l'entropie approximée (ApEn) et échantillonnée (SampEn), une diminution de la variabilité du rythme cardiaque et des décélérations transitoires (21-24). Ce monitoring des caractéristiques du rythme cardiaque est simple, non-invasif et rapidement disponible (il ne nécessite que 30 minutes d'enregistrement de l'électrocardiogramme néonatal). Il est déjà utilisé en pratique courante en néonatologie et a démontré son intérêt dans la diminution de la mortalité chez les enfants de faible poids (25).

En vue d'optimiser l'analyse de la VRC fœtal et par analogie avec l'adulte et le nouveau-né, l'acquisition de l'ECG fœtal de manière non invasive a fait l'objet de nombreux travaux ces dernières années. Cette technique est difficile du fait de la taille cardiaque fœtale et par la nécessité d'un traitement important du signal pour dissocier l'ECG fœtal de l'ECG maternel. Des travaux récents montrent qu'une analyse de la VRC n'est possible que sur moins de 10% de l'enregistrement voire quasiment impossible entre 30-34 SA du fait de la présence du *vernix caseosa* qui isole électriquement le fœtus causant une atténuation importante du signal ECG fœtal (26). Ainsi, l'outil le plus couramment utilisé en obstétrique reste le recueil du signal cardiaque par ultrasons. L'enregistrement du rythme cardiaque fœtal (ERCF) est obtenu par un recueil doppler de la fréquence cardiaque fœtale en moyennant plusieurs cycles cardiaques fœtaux.

Au cours de la grossesse, l'étude visuelle de la VRC fœtal est un des critères majeurs de l'analyse de l'ERCF, examen de première intention pour l'évaluation du bien-être fœtal, en particulier dans la surveillance des RPM avant terme. Néanmoins, l'interprétation visuelle de l'ERCF n'est pas fiable, en dehors des rythmes strictement normaux et de ceux hautement pathologiques avec de nombreux ralentissements. Plusieurs études ont démontré l'étendue des variations inter-observateurs, mais également intra-observateurs, dans l'analyse de l'ERCF (27,28).

Une analyse informatisée de l'ERCF, tentant une approche objective de la VRC fœtal via la mesure de différents paramètres, a donc été élaborée et commercialisée depuis 1989 sous la forme du logiciel OXFORD® 8000 puis 8002 (29). Elle permet le calcul de :

- la fréquence cardiaque de base : correspond au rythme cardiaque fœtal moyen pendant le tracé, sans accélération ni décélération
- les accélérations et décélérations : déviations par rapport à la ligne de base
- les épisodes de haute et de basse variation : ces épisodes sont définis comme toute partie de l'enregistrement où, par rapport à la ligne de base, la variation de l'amplitude sur une minute est supérieure à 32 ms (haute variation) ou inférieure à 30 ms (basse variation) pendant 5 à 6 minutes consécutives. Les épisodes de haute variation sont associés au sommeil actif du fœtus tandis que les épisodes de basse variation sont associés au sommeil calme.
- la variation à court terme (VCT) : mesure des « micro » fluctuations du rythme cardiaque fœtal et ne peut être mesurée à l'œil nu. Elle est indépendante de la ligne de base. La VCT est mesurée en divisant chaque minute du tracé en 16 sections de 3,75 secondes. L'intervalle de pulsation moyen de chaque section est calculé et le changement de ces valeurs moyennes d'une section à l'autre détermine la VCT.

Notre précédente étude monocentrique réalisée au CHU de Rennes (annexe 2, données encore non soumises à publication dans l'attente d'un dépôt de brevet) sur une cohorte de 23 patientes avec une RPM avant terme a permis de montrer pour la première fois que l'analyse de la variabilité du rythme cardiaque fœtal (RCF) via un recueil doppler du signal cardiaque est une voie de recherche prometteuse pour le dépistage de la chorioamniotite.

En effet, on observe une modification de certains paramètres de variabilité cardiaque du fœtus en cas de chorioamniotite prouvée histologiquement. Parmi ces modifications, on retrouve une augmentation de la fréquence cardiaque de base (p=0,02), une augmentation des épisodes de basse variation (p=0,04), une diminution de la variation à court terme (p=0,003) et des épisodes de haute variation (p < 0,001) dans les derniers enregistrements réalisés avant la naissance (spontanée ou provoquée) en cas de chorioamniotite. L'indice des épisodes de haute variation (rapport entre la moyenne du nombre d'épisodes de haute variation des deux derniers enregistrements et les quatre précédents) apparaît comme un outil prometteur pour le diagnostic précoce de la chorioamniotite dans les RPM avant terme (sensibilité 90%, spécificité 84,6%, valeur prédictive positive 71,5%, valeur prédictive négative 95,2%, aire sous la courbe = 0,88, intervalle de confiance à 95% 0,73-100). Ces données vont dans le sens de celles observées en cas d'infection néonatale et sont cohérentes avec les mécanismes physiopathologiques sous-jacents (perte de la variabilité, diminution de l'adaptabilité fœtale en réaction au stimulus infectieux/inflammatoire placentaire).
Ces résultats incitent donc à la réalisation d'une étude prospective afin de tester cet indice et de confirmer les résultats sur l'ensemble du recueil du signal cardiaque réalisé au cours de la surveillance des RPM avant terme.

Conclusion

Le dépistage précoce de la chorioamniotite dans les RPM avant terme est un enjeu majeur de la recherche en obstétrique et il manque encore aujourd'hui un outil diagnostique efficace. Si les résultats de notre étude pilote étaient confirmés, l'intégration de ces marqueurs de la variabilité cardiaque dans un monitoring temps-réel répondrait à cet objectif. Un tel dispositif, simple, facilement accessible, reproductible et non invasif, fournirait au clinicien un outil d'aide à la décision clinique (extraction fœtale) qui permettrait une diminution de la morbi/mortalité néonatale en évitant de surajouter les complications de la chorioamniotite à celles de la prématurité lors de la naissance en cas de RPM avant terme.

3. OBJECTIFS

3.1. Objectif principal

L'objectif principal est de mesurer la valeur diagnostique de l'indice des épisodes de haute variation du rythme cardiaque fœtal (rapport entre la moyenne du nombre d'épisodes de haute variation des deux derniers enregistrements et les quatre précédents) pour la détection de la chorioamniotite prouvée histologiquement dans les RPM avant terme.

3.2. Objectif(s) secondaire(s)

Les objectifs secondaires sont :

- caractériser l'évolution des paramètres de la variabilité cardiaque fœtale **et néonatale (analyse linéaire et non linéaire, cf annexe 1) sur une population de RPM avant terme avec et sans chorioamniotite histologique.** tester l'hypothèse que les autres paramètres d'analyse de la variabilité cardiaque fœtale peuvent constituer, seuls ou en association notamment avec l'indice des épisodes de haute variation, un indicateur uni ou multivarié de la chorioamniotite histologique dans les RPM avant terme.

4. DEFINITION DES SUJETS ELIGIBLES

4.1. Critères d'inclusion

Les critères d'inclusion sont :

- patiente majeure
- grossesse singleton
- RPM survenue entre 26 et 34 SA, authentifiée à l'examen clinique et si doute, confirmée par un test diagnostique vaginal détectant l'IGFBP-1.
- patiente ayant reçu l'information sur le protocole et n'ayant pas manifesté son opposition à participer

4.2. Critères de non inclusion

Les critères de non inclusion sont :

- grossesse multiple
- hypotrophie néonatale (poids de naissance <10ème percentile AUDIPOG)
- tabagisme maternel actif
- diabète gestationnel ou préexistant à la grossesse
- pathologie maternelle :
    - cardiopathie congénitale ou acquise
    - embolie pulmonaire en cours de traitement
    - hypertension artérielle pulmonaire
    - insuffisance rénale chronique modérée à sévère
    - Bronchopneumopathie chronique obstructive
    - Maladie auto-immune (lupus érythémateux disséminé, sclérose en plaques, syndrome de Gougerot-Sjögren)
- malformation fœtale avec anomalie cardiaque, neurologique ou génétique avérée

4.3. Critère d'exclusion pour l'objectif principal

Accouchement dans les 48 heures suivant la RPM (situation clinique ne permettant pas le calcul de l'indice des épisodes de haute variation ni de modéliser l'évolution des paramètres d'analyse de la VRC)

5. CRITERES DE JUGEMENT

5.1. Critère principal

Le critère de jugement principal est l'indicateur d'efficacité (aire sous courbe ROC, sensibilité, spécificité, valeur prédictive positive, valeur prédictive négative) de l'indice des épisodes de haute variation pour la détection de la chorioamniotite histologique dans les RPM avant terme.

5.2. Critère(s) secondaire(s)

- Les performances de l'approche seront aussi testées par les changements observés dans l'évolution des paramètres de la variabilité cardiaque fœtale et néonatale (analyse linéaire et non linéaire). L'expression quantitative (analyse statistique) et modélisée (analyse graphique et statistique) de l'évolution de ces

marqueurs permettra de définir leur valeur diagnostique pour le diagnostic de chorioamniotite histologique et/ou d'infection materno-fœtale dans les RPM avant terme.

- un indicateur d'efficacité (aire sous courbe ROC, sensibilité, spécificité, valeur prédictive positive, valeur prédictive négative) sera aussi utilisé pour évaluer la valeur diagnostique des autres paramètres d'analyse de la variabilité cardiaque retenus sous la forme d'indicateur soit uni ou multivarié pour la détection de la chorioamniotite histologique dans les RPM avant terme.

6. IDENTIFICATION DU DISPOSITIF MÉDICAL

Le recueil doppler du signal cardiaque fœtal sera effectué à l'aide d'un moniteur cardiotocographique F3 Fetal Monitor (EDAN Instruments, Inc ; figure 1) qui dispose des caractéristiques techniques communes à tous les cardiotocographes concurrents. Ce dispositif a l'avantage d'être muni d'une mémoire interne de 60 heures permettant de stocker les enregistrements effectués sous forme de fichiers TRC (Trace file) indépendants, transférables sur ordinateur via un port USB. Ces fichiers TRC permettent d'obtenir sous forme numérique le relevé des battements cardiaques fœtaux rééchantillonnés à 4 Hz et de procéder ainsi au calcul des paramètres de variabilité cardiaque sous Matlab® (Mathworks Inc.).

Le recueil du signal cardiaque néonatal par ECG sera réalisé 48h après la naissance, puis 1 fois par semaine grâce aux extraits du signal ECG issu du moniteur de surveillance polygraphique : séries brutes et rééchantillonnées à 4

Hz de durées des cycles cardiaques successifs (RR), axe morphologie de QRS et P.

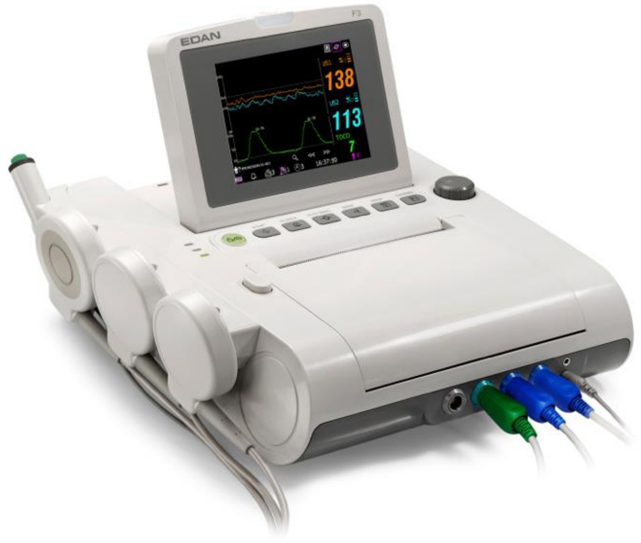

Figure 1 : Moniteur F3 (EDAN Instruments, Inc)

7. CONCEPTION DE LA RECHERCHE

7.1. Méthodologie de la recherche

Etude contrôlée cas-témoins multicentrique d'observation avec analyse en insu du personnel soignant.

7.2. Déroulement de la recherche

L'inclusion sera prospective : toute patiente enceinte d'une grossesse singleton hospitalisée pour RPM survenue entre 26 et 34 SA sera potentiellement incluse dans l'étude.

Le recueil doppler du signal cardiaque sera prospectif dès l'inclusion, extrait des enregistrements doppler réalisés dans le cadre de la surveillance habituelle du bien être fœtal. L'analyse des paramètres de variabilité cardiaque (indice des épisodes de haute variation, analyse linéaire et non linéaire) sera réalisée à l'aide du logiciel Matlab® en insu du personnel soignant. Les algorithmes de calcul de ces paramètres ont déjà été développés par le Laboratoire Traitement du Signal et de l'Image.

Le recueil des données cliniques sera prospectif dans une base de données d'événements relationnels. Il concernera les données maternelles, placentaires et néonatales.

Les données doppler des signaux cardiaques fœtaux, préalablement anonymisées, seront stockées au fur et à mesure sur un ordinateur dédié à l'étude. Une copie des signaux acquis sera réalisée régulièrement sur le serveur

sécurisé du CIC-IT via le dispositif ASCENT (Anonymised System for Clinical experimentation : https://ascent.univ-rennes1.fr/) et stockée au fur et à mesure sur une base de données dédiée à l'étude.
Par ailleurs, un protocole commun a été établi avec les centres investigateurs fixant notamment les modalités des enregistrements du rythme cardiaque fœtal. Ce protocole ne modifie pas significativement les pratiques actuelles établies dans ces centres qui sont déjà harmonisées.

Sélection des participantes
La sélection des patientes sera effectuée au sein du service de Gynécologie-Obstétrique du CHU de Rennes, Angers, Nantes, Poitiers.

Visite d'inclusion (=J0)
Elle a pour but de présenter les objectifs de l'étude au patient, de vérifier les critères d'inclusion et de non inclusion et de recueillir la non-opposition de la patiente.
Lors de cette visite les données suivantes sont recueillies :

- données cliniques maternelles
    - Age
    - Taille et poids avec calcul de l'indice de masse corporelle
    - Datation précise de la grossesse (échographie obstétricale réalisée entre 11 SA + 1j et 14 SA + 6j)
    - Antécédents obstétricaux : parité, pathologie obstétricale antérieure (RPM avant terme, prééclampsie, retard de croissance intra-utérin, accouchement prématuré), mode d'accouchement (voie basse, voie basse instrumentale, césarienne)
    - Terme de la rupture prématurée des membranes et couleur du liquide amniotique
- données fœtales
    - ERCF informatisé initial

RPM-naissance
Lors de ces visites les données suivantes sont recueillies :

- données maternelles :
    - données biologiques : numérations leucocytaires, dosages de la CRP, résultats des prélèvements vaginaux bactériologiques**,** données cliniques : température corporelle, couleur du liquide amniotique
    - date et heure des injections de CELESTENE®
    - Mise en place d'une tocolyse (ADALATE®, TRACTOCILE®) : mode d'administration, dates et durée du/des traitement(s)
    - Antibiothérapie : antibiotique(s), dates et durée du/des traitement(s)
- données fœtales:
    - ERCF : sauvegardés sur le moniteur, sont transférés de manière hebdomadaire via une clé USB et transmis par Internet au Laboratoire Traitement du Signal et de l'Image (LTSI-UMR INSERM 1099) via le site sécurisé du dispositif ASCENT (https://ascent.univ-rennes1.fr/)

- Oligoamnios ou anamnios

Naissance

Lors de cette visite les données suivantes sont recueillies :

- mode d'accouchement et motif (en particulier, suspicion de chorioamniotite clinique) sauf si travail spontané: travail spontané, déclenchement, césarienne en urgence
- contexte de survenue du travail : fièvre maternelle, modifications du liquide amniotique, contractions utérines, anomalies de l'ERCF (visuelle ou informatisée)
- Voie d'accouchement : voie basse, voie basse instrumentale, césarienne
- Poids de naissance (exprimé en valeur absolue et en percentile selon les courbes de références AUDIPOG)
- pH artériel et lactates au cordon ombilical, base excess, PCO2,
- Mode de sortie de l'enfant (réanimation, soins courants, soins intensifs, suites de couches)
- Décès néonatal : date, heure et résultat autopsique si réalisé

Période post-natale(2j à 15 semaines)

Lors de cette période les données suivantes sont recueillies :

- dans les 72 heures suivant la naissance :
    - données biologiques néonatales : CRP (toutes), Procalcitonine, prélèvements bactériologiques périphériques et hémocultures, glycémie (aléatoire)
    - données cliniques néonatales :
        - respiratoires :-surfactant, mode de ventilation
        - cardiovasculaires :-HTAP, utilisation d'amines vasopressines
        - sanguines : anémie (hemoglobine), ictère (asat, alat, bilirubine directe et conjuguée)
        - évaluation de la suspicion d'infection materno-fœtale : certaine, probable ou possible selon les critères du National Institute for Health and Care Excellence (NICE guidelines CG149)
        - digestif/hépatobiliaire : entérocolite ulcéro-nécrosante avec stade de Bell modifié

    - données de variabilité cardiaque : sauvegarde de l'ECG néonatal, (1 enregistrement d'une heure dans les 48 premières heures, puis une fois par semaine jusqu'à la sortie d'hospitalisation)

- à distance :
    - examen anatomopathologique du placenta sur grille standardisée collectant les informations visant à l'identification des chorioamniotites (25,26)
        - chorioamniotite aigüe (inflammation maternelle)
            - stade 1 : infiltrat à polynucléaires neutrophiles (PNN) dans le toit de la chambre intervilleuse
            - stade 2 : stade 1 + infiltrat à PNN dans la plaque choriale

        - stade 3 : stade 2 + nécrose des PNN et/ou des cellules amniotiques
      - réaction inflammatoire fœtale
        - stade 1 : vaisseaux de la plaque choriale et/ou veine ombilicale
        - stade 2 : stade 1 + artère ombilicale
        - stade 3 : stade 2 + diffusion dans la gelée de Wharton
      - présence d'infarctus placentaires (anciens et/ou récents) avec pourcentage de la surface placentaire concernée (<10%, entre 10 et 30%, > 30%)
      - présence d'un hématome rétro-placentaire
      - présence d'un hématome décidual marginal
  - bilan des pathologies néonatales cérébrales (résultats de l'imagerie cérébrale : ETF et IRM)

**Actions**	J0<br>(Visite d'inclusion à l'admission)	Intervalle libre RPM/naissance<br>(RPM à 15 semaines)	Naissance	Période post-natale<br>(néonatologie suivi de 15 semaines maximum)
Signature de la non opposition	X			
Fax d'inclusion	X			
Données cliniques materno-fœtales	X	X		
Recueil des ERCF informatisés	X	X		
Données cliniques néonatales			X	X
Examen anatomopathologique du placenta				X
Recueil des ECG			X	X

8. NOMBRE DE SUJETS NECESSAIRE

La prévalence de la chorioamniotite histologique au cours des RPM avant terme est de 20 à 30% dans la littérature (1,10). En sachant que la moyenne de l'indice des épisodes de haute variation (annexe 2, tableau 1) est en valeur absolue de 3.82 en cas de chorioamniotite et de 1.18 en l'absence de chorioamniotite avec un écart type commun de 1.7, un échantillon de 60 patientes analysables a une puissance de 95 % pour détecter une différence significative en formulation bilatérale et en utilisant un test t de Student avec un p significatif inférieur à 0.05 en se basant sur une prévalence de 15 % de chorioamniotite histologique. Compte tenu du critère d'exclusion pour l'analyse de l'objectif principal (accouchement dans les 48 heures suivant la RPM qui concerne 50% des RPM avant terme) un échantillon de 120 patientes dont 60 patientes analysables pour l'objectif principal est nécessaire. En tenant compte d'une marge de sécurité de 30 % (refus de participation ou présence de critères de non inclusion), cette étude nécessite un potentiel d'inclusion minimal de 160 patientes.

Le recrutement envisagé est réaliste. Les effectifs par an de RPM avant terme aux CHU de Rennes, Angers, Nantes

et Poitiers peuvent être estimés respectivement à 100, 100, 100 et 60.

9. ANALYSE STATISTIQUE

L'estimation de la capacité prédictive de l'indice des épisodes de haute variation ainsi que l'indicateur uni/multivarié obtenu à partir des paramètres de variabilité cardiaque se fera sur les critères de sensibilité, spécificité, taux de faux-positifs et faux-négatifs, aire sous la courbe ROC.

Une analyse exploratoire univariée des paramètres d'analyse de la variabilité cardiaque sera réalisée. Des comparaisons de distributions, de moyennes et médianes de ces paramètres dans les blocs de monitoring associés à une chorioamniotite histologique et dans les blocs non associés à une chorioamniotite histologique seront effectuées (tests *t* de Student et Mann-Whitney).

L'évolution au cours du temps des différents paramètres mesurés sera comparée entre les deux populations par une ANOVA pour temps répétés à 2 facteurs (âge gestationnel et chorioamniotite). L'effet sujet sera inclus dans le modèle pour tenir compte du caractère apparié des mesures.

10. FAISABILITE DU PROJET

La faisabilité de ce projet repose sur un environnement privilégié associant les compétences complémentaires de plusieurs entités :

1/ Le **Laboratoire Traitement du Signal et de l'Image (LTSI – UMR INSERM 1099)** qui apporte les connaissances sur le recueil, le traitement, l'analyse du signal ainsi qu'une expertise de longue date dans la maitrise et la mise au point de monitorings médicaux. L'avancée des travaux de l'équipe SEPIA du LTSI, sur la détection d'infection des grands prématurés est en cours d'évaluation dans le cadre du PHRC Caress-Premi, et constitue une base solide à la réussite de ce projet.

Avec le soutien du Groupement Interrégional de Recherche Clinique et d'Innovation des hôpitaux Universitaires (GIRCI) du Grand Ouest (projet PhysioDev), le LTSI est aussi à l'origine de la conception et du développement du dispositif sécurisé d'anonymisation et de stockage de données ASCENT (https://ascent.univ-rennes1.fr/) qui garantit la transmission des données issues des ERCF dans des conditions optimales.

2/ Ce projet bénéficie du soutien du **réseau HUGOPEREN** (Hôpitaux Universitaires du Grand Ouest PEdiatric REsearch Network), financé par le GIRCI du Grand Ouest, avec les services de **Gynécologie-Obstétrique des CHU de Rennes** (>4000 accouchements/an potentiel d'inclusion : 100 RPM avant terme/an), d'**Angers** (> 4000 accouchements/an, potentiel d'inclusion : 100 RPM avant terme/an), de **Nantes** (> 4000 accouchements/an, potentiel d'inclusion : 100 RPM avant terme/an) et de **Poitiers** (> 2500 accouchements/an, potentiel d'inclusion : 60 RPM avant terme/an) qui garantissent le recrutement nécessaire à cette étude.

3/ Le **Centre d'Investigation Clinique et d'Innovation Technologique (CIC 1414) du CHU de Rennes** qui met à disposition son plateau technique et son savoir-faire en validation d'algorithmes de traitement du signal et de dispositifs médicaux.

11. ASPECTS LOGISTIQUES, LEGAUX ET GENERAUX

11.1. Calendrier prévisionnel

Durée de la période d'inclusion : 39 mois
Durée maximale de la participation pour chaque couple mère/bébé : environ 19 semaines (5 mois)
Durée de traitement des données et rapports : 6 mois
Durée maximale totale de l'étude : 50 mois

11.2. Comité d'éthique

Le responsable de l'étude, le CHU de Rennes, soumettra avant toute mise en œuvre de la recherche pour avis, le protocole d'étude, la lettre d'information et de non opposition au comité d'éthique de Rennes.

11.3. CNIL

Cette étude entre dans le cadre de la « Méthodologie de Référence » (MR-003) en application des dispositions de l'article 54 alinéa 5 de la loi n°78-17 du 6 janvier 1978 modifiée relative à l'informatique, aux fichiers et aux libertés. Le CHU de Rennes, promoteur de l'étude, a signé un engagement de conformité à cette « Méthodologie de Référence ».

11.4. Information et non opposition

Les patientes seront informées de façon complète et loyale en des termes compréhensibles, des objectifs de l'étude, de leurs droits de refuser de participer à l'étude ou de la possibilité de se rétracter à tout moment y compris pour leur enfant. Toutes ces informations figureront sur une lettre d'information et de non opposition remis aux patientes.

Tout ou partie des données recueillies à l'occasion de ce protocole de recherche pourront faire l'objet d'une cession à d'autres chercheurs en France ou à l'étranger dans la mesure où le sujet n'aura pas exercé son droit d'opposition.

11.5. Modifications substantielles

Toute modification substantielle au protocole de l'étude devra être notifiée aux autorités compétentes.

11.6. Confidentialité des données

Les personnes ayant un accès direct prendront toutes les protections nécessaires en vue d'assurer la confidentialité des informations relatives aux personnes qui s'y prêtent et notamment en ce qui concerne leur identité ainsi qu'aux résultats obtenus.
Ces personnes, au même titre que les investigateurs eux-mêmes, sont soumises au secret professionnel (selon les conditions définies par les articles 226-13 et 226-14 du code pénal).
Pendant la recherche ou à son issue, les données recueillies sur les personnes qui s'y prêtent et transmises au responsable de l'étude par les investigateurs (ou tout autre intervenant spécialisé) seront rendues anonymes.
Elles ne doivent en aucun cas faire apparaître en clair les noms des personnes concernées ni leur adresse.
Seule la première lettre du nom du sujet et la première lettre de son prénom seront enregistrées, accompagnées

d’un numéro codé propre à l’étude indiquant l’ordre d’inclusion des sujets.

11.7. Contrôle et assurance de la qualité

Un Attaché de Recherche Clinique (ARC) mandaté par le responsable de l’étude s’assurera de la bonne réalisation de l’étude, du recueil des données générées par écrit, de leur documentation, enregistrement et rapport conformément aux dispositions législatives et réglementaires en vigueur, relatives à la conduite d’une étude non interventionnelle.

Chaque investigateur et les membres de son équipe acceptent de se rendre disponibles lors des visites de Contrôle de Qualité effectuées à intervalles réguliers par l’Attaché de Recherche Clinique. Lors de ces visites, les éléments suivant seront revus :

- suivi des inclusions
- respect du protocole de l'étude et des procédures qui y sont définies.

Les investigateurs s’engagent à accepter les éventuels audits d’assurance qualité effectués par le responsable de l’étude ainsi que les inspections effectuées par les Autorités Compétentes. Toutes les données, tous les documents et rapports peuvent faire l'objet d'audits et d'inspections réglementaires sans que puisse être opposé le secret médical.

11.8. Recueil des données

Toutes les informations requises par le protocole doivent être consignées dans les cahiers d’observation. Les données devront être recueillies au fur et à mesure qu'elles sont obtenues, et enregistrées dans ces cahiers de façon explicite. Chaque donnée manquante devra être codée.

Ce cahier d’observation électronique sera mis en place dans chacun des centres grâce à un support Internet de recueil des données. Un document d’aide pour l’utilisation de cet outil sera fourni aux investigateurs.

Le remplissage du cahier d'observation via internet par l'investigateur permet ainsi à l'ARC de visualiser rapidement et à distance les données. L'investigateur est responsable de l'exactitude, de la qualité et de la pertinence de toutes les données saisies. De plus, lors de leurs saisies, ces données sont immédiatement vérifiées grâce à des contrôles de cohérence. A ce titre, il doit valider toute modification de valeur dans le CRF. Ces modifications font l'objet d'un audit trail. Une justification peut éventuellement être intégrée en commentaire. Une impression papier sera demandée en fin d'étude, authentifiée (datée et signée) par l'investigateur. Une copie du document authentifié à destination du promoteur devra être archivée par l’investigateur.

Les données doppler des signaux cardiaques fœtaux, et les enregistrements ECG préalablement anonymisées, seront stockés au fur et à mesure sur un ordinateur dédié à l’étude. Une copie des signaux acquis sera réalisée de façon hebdomadaire via port USB depuis les moniteurs puis transmis sur le serveur sécurisé du CIC-IT via le dispositif ASCENT (Anonymised System for Clinical experimentation : https://ascent.univ-rennes1.fr/) et stockée au fur et à mesure sur une base de données localisée sur un ordinateur dédié spécifiquement à l’étude.

11.9. Archivage

Les documents suivants seront conservés dans les services respectifs jusqu’à la fin de la période d’utilité pratique.

Ces documents sont :
- Protocole et annexes, modifications substantielles éventuelles,
- Données individuelles (copies authentifiées de données brutes)
- Documents de suivi
- Analyses statistiques
- Rapport final de l'étude

Les documents de l'étude devront être archivés par le responsable de l'étude pendant une durée minimum de 15 ans.

Aucun déplacement ou destruction ne pourra être effectué sans l'accord du responsable de l'étude. Au terme des 15 ans, le responsable de l'étude sera consulté pour destruction. Toutes les données, tous les documents et rapports pourront faire l'objet d'audit ou d'inspection.

11.10. Assurance

Le CHU de Rennes est le responsable de l'étude, sans nécessité de souscrire une assurance pour cette étude non interventionnelle.

11.11. Règles relatives à la publication

Les règles de publication sont les suivantes :

- signature par le porteur du projet puis par ceux qui viendraient apporter une contribution significative au cours du déroulement de l'étude ;
- mention de l'origine du financement.

En cas d'études annexes, les résultats de celles-ci ne pourront être publiés qu'avec l'accord du porteur du projet et uniquement après publication de l'étude principale qui devra être citée.

12. BIBLIOGRAPHIE

1. Redline RW, Faye-Petersen O, Heller D, Qureshi F, Savell V, Vogler C, et al. Amniotic infection syndrome: nosology and reproducibility of placental reaction patterns. Pediatr Dev Pathol. 2003 Sep;6(5):435–48.
2. Gallot D, Guibourdenche J, Sapin V, Goffinet F, Doret M, Langer B, et al. Which biological test to confirm rupture of membranes? J Gynecol Obstet Biol Reprod. 2012 Apr;41(2):115–21.
3. Parry S, Strauss JF. Premature rupture of the fetal membranes. N Engl J Med. 1998 Mar 5;338(10):663–70.
4. Simhan HN, Canavan TP. Preterm premature rupture of membranes: diagnosis, evaluation and management strategies. BJOG. 2005 Mar;112 Suppl 1:32–7.
5. Goldenberg RL, Culhane JF, Iams JD, Romero R. Epidemiology and causes of preterm birth. Lancet. 2008 Jan 5;371(9606):75–84.
6. Aziz N, Cheng YW, Caughey AB. Neonatal outcomes in the setting of preterm premature rupture of membranes complicated by chorioamnionitis. J Matern Fetal Neonatal Med. 2009 Sep;22(9):780–4.
7. Ramsey PS, Lieman JM, Brumfield CG, Carlo W. Chorioamnionitis increases neonatal morbidity in pregnancies complicated by preterm premature rupture of membranes. Am J Obstet Gynecol. 2005

Apr;192(4):1162–6.
8. Dammann O, Leviton A, Gappa M, Dammann CEL. Lung and brain damage in preterm newborns, and their association with gestational age, prematurity subgroup, infection/inflammation and long term outcome. BJOG. 2005 Mar;112 Suppl 1(s1):4–9.
9. Wu YW. Systematic review of chorioamnionitis and cerebral palsy. Ment Retard Dev Disabil Res Rev. 2002;8(1):25–9.
10. Yoon BH, Romero R, Park JS, Kim CJ, Kim SH, Choi JH, et al. Fetal exposure to an intra-amniotic inflammation and the development of cerebral palsy at the age of three years. Am J Obstet Gynecol. 2000 Mar;182(3):675–81.
11. Spinillo A, Capuzzo E, Stronati M, Ometto A, Orcesi S, Fazzi E. Effect of preterm premature rupture of membranes on neurodevelopmental outcome: follow up at two years of age. BJOG. 1995;102(11):882–7.
12. Dammann O, Kuban KCK, Leviton A. Perinatal infection, fetal inflammatory response, white matter damage, and cognitive limitations in children born preterm. Ment Retard Dev Disabil Res Rev. 2002;8(1):46–50.
13. Yoon BH, Park C-W, Chaiworapongsa T. Intrauterine infection and the development of cerebral palsy. BJOG. 2003 Apr;110 Suppl 20:124–7.
14. Pacora P, Chaiworapongsa T, Maymon E, Kim YM, Gomez R, Yoon BH, et al. Funisitis and chorionic vasculitis: the histological counterpart of the fetal inflammatory response syndrome. J Matern Fetal Neonatal Med. 2002 Jan;11(1):18–25.
15. Collège des Gynécologues et Obstétriciens Français (CNGOF). Rupture prématurée des membranes : recommandations pour la pratique clinique. wwwcngofassofr. (1999).
16. American College of Obstetricians and Gynecologists. Practice Bulletins No. 139. Obstet Gynecol. 2013 Oct;122(4):918–30
17. Audibert F. Diagnosis of infection in the case of premature rupture of the membranes. J Gynecol Obstet Biol Reprod. 1999 Nov;28(7):635-41
18. Popowski T, Goffinet F, Batteux F, Maillard F, Kayem G. Prediction of maternofetal infection in preterm premature rupture of membranes: serum maternal markers. Gynecol Obstet Fertil. 2011 May;39(5):302–8.
19. Xhyheri B, Manfrini O, Mazzolini M, Pizzi C, Bugiardini R. Heart rate variability today. Prog Cardiovasc Dis. 2012 Nov;55(3):321–31.
20. Pincus SM, Cummins TR, Haddad GG. Heart rate control in normal and aborted-SIDS infants. Am J Physiol. 1993 Mar;264(3 Pt 2):R638–46.
21. Griffin MP, O'Shea TM, Bissonette EA, Harrell FE, Lake DE, Moorman JR. Abnormal heart rate characteristics are associated with neonatal mortality. Pediatr Res. 2004 May;55(5):782–8.
22. Griffin MP, O'Shea TM, Bissonette EA, Harrell FE, Lake DE, Moorman JR. Abnormal heart rate characteristics preceding neonatal sepsis and sepsis-like illness. Pediatr Res. 2003 Jun;53(6):920–6.
23. Griffin MP, Lake DE, Bissonette EA, Harrell FE, O'Shea TM, Moorman JR. Heart rate characteristics: novel physiomarkers to predict neonatal infection and death. Pediatrics. 2005 Nov;116(5):1070–4.
24. Beuchée A, Carrault G, Bansard JY, Boutaric E, Bétrémieux P, Pladys P. Uncorrelated randomness of the heart rate is associated with sepsis in sick premature infants. Neonatology. 2009;96(2):109–14.

25. Moorman JR, Carlo WA, Kattwinkel J, Schelonka RL, Porcelli PJ, Navarrete CT, et al. Mortality reduction by heart rate characteristic monitoring in very low birth weight neonates: a randomized trial. J Pediatr. 2011 Dec;159(6):900–1.
26. van Laar JOEH, Warmerdam GJJ, Verdurmen KMJ, Vullings R, Peters CHL, Houterman S, et al. Fetal heart rate variability during pregnancy, obtained from non-invasive electrocardiogram recordings. Acta Obstet Gynecol Scand. 2013 Oct 17;93(1):n/a–n/a.
27. Bernardes J, Costa-Pereira A, Ayres-de-Campos D, van Geijn HP, Pereira-Leite L. Evaluation of interobserver agreement of cardiotocograms. Int J Gynaecol Obstet. 1997 Apr;57(1):33–7.
28. Gagnon R, Campbell MK, Hunse C. A comparison between visual and computer analysis of antepartum fetal heart rate tracings. Am J Obstet Gynecol. 1993 Mar;168(3 Pt 1):842–7.
29. Dawes GS, Moulden M, Redman CW. System 8000: computerized antenatal FHR analysis. J Perinat Med. 1991;19(1-2):47–51.

13. LISTE DES ANNEXES

Annexe 1 : L'analyse de la variabilité cardiaque

Annexe 2 : Analyse informatisée du rythme cardiaque fœtal et dépistage précoce de la chorioamniotite dans les ruptures prématurées des membranes (résumé de l'étude rétrospective menée au CHU de Rennes)

Annexe 3 : Lettre d'information et de non opposition

Annexe 4 : Protocole commun de prise en charge
