# Supplementary material for: Day-to-day dynamics of fetal heart rate variability to detect chorioamnionitis in preterm premature rupture of membranes
Source: PLoS One. 2025 Jan 2;20(1):e0305875. doi: 10.1371/journal.pone.0305875 (PMC11695014; doi:10.1371/journal.pone.0305875)
Supplement: S2 File — (PDF) [file pone.0305875.s003.pdf]

Computerized analysis of fetal heart rate variability for early detection of chorioamnionitis in preterm premature rupture of membranes

Study coordinator: CHU de Rennes, Hôpital de Pontchaillou, 2 rue Henri le Guilloux, 35033 Rennes cedex 9, France

Principal investigator: Dr Linda Lassel
Department of Gynecology-Obstetrics and Reproductive Medicine, CHU de Rennes, Hôpital Sud, 16 boulevard de Bulgarie, 35203 Rennes cedex 2, France
SEPIA Unit - Signal and Image Processing Laboratory (LTSI, UMR INSERM 1099), University of Rennes 1, Campus de Beaulieu, Bâtiment 22, 35042 Rennes cedex
Tel : 06.34.18.25.88 - Email : linda.lassel@chu-rennes.fr

Methodologist:
Dr Bruno Laviolle, Clinical Investigation Centre, Inserm 1414, Clinical Pharmacology Unit, Pontchaillou Hospital, 2 rue Henri le Guilloux, 35033 Rennes cedex 9. Tel: 02.99.28.96.68 - Email: bruno.laviolle@chu-rennes.fr

Monitoring: Direction de la Recherche Clinique, Hôpital de Pontchaillou, 2 rue Henri le Guilloux, 35033 Rennes cedex 9. Tel: 02.99.28.25.55 - Email: drc@chu-rennes.fr

Version history
Version submitted to the CER: 1.0 of 14 October 2014
Version accepted by the CER: 2.0 of 17 November 2014
Version sent for information: 4.0 of 8 December 2014
Version submitted for modification no. 2: 5.4 of 16 January 2017
Modified version accepted by the CER: 6.0 of 21/01/2017
Version submitted for modification no. 3: 7.0 of 23/02/2018
Modified version accepted by the CER: 8.0 of 20/04/2018
Version sent for modification no. 3: 7.0 of 23/02/2018
Modified version accepted by the CER: 8.0 of 20/04/2018
Version submitted for modification no. 4: 9.0 of 31/08/2018
Modified version accepted by the CER: 10.0 of 25/09/2018
Version submitted for modification no. 5: 11.0 of 16/11/2020
Modified version accepted by the CER: 12.0 of 17/11/2020
Modified version sent for information to CER no. 6: 14.0 of 08/11/2021

RESPONSIBLE FOR THE STUDY:	CHU DE RENNES
**CLINICAL TRIAL PROTOCOL**<br>**AiRPM**	
**TEST CODE**	35RC14_9772
**FULL TITLE**	Computerized analysis of fetal heart rate variability for early detection of chorioamnionitis in preterm premature rupture of membranes
**INDICATION(S) (TARGET)**	Premature rupture of membranes before term
**PRINCIPAL INVESTIGATOR**	Dr Linda Lassel<br>Department of Gynecology-Obstetrics and Reproductive Medicine<br>Rennes University Hospital<br>Hôpital Sud<br>16 Boulevard de Bulgarie<br>35203 Rennes cedex 2
**PROTOCOL VERSION NUMBER**	Version 14.0
**DATE OF PROTOCOL**	8 November 2021
**CER**	*Notice date: 10 November 2014*<br>*Advisory no.: 14.76*
**CNIL**	*Declaration date: 17October 2014*<br>*Declaration no.: 1802940 v 0*<br>*MR003 compliance undertaking: 7 November 2016*
**THIS CONFIDENTIAL DOCUMENT IS THE PROPERTY OF CHU DE RENNES**<br>**NO UNPUBLISHED INFORMATION CONTAINED IN THIS DOCUMENT MAY BE DIVULGED WITHOUT PRIOR WRITTEN AUTHORISATION FROM CHU DE RENNES**	

CONTENTS

1. GENERAL INFORMATION 14
  1.1. Title 14
  1.2. Manager 14
  1.3. Coordination and monitoring of the study 14
  1.4. Principal investigator 14
  1.5. investigator(s) 14
  1.6. Associate scientists 15
  1.7. Methodologist 16
2. RATIONALE FOR THE STUDY 16
3. OBJECTIVES 19
  3.1. Main objective 19
  3.2. Secondary objective(s) 19
4. DEFINITION OF ELIGIBLE SUBJECTS 19
  4.1. Inclusion criteria 19
  4.2. Non-inclusion criteria 19
  4.3. Exclusion criteria for the main objective 20
5. JUDGING CRITERIA 20
  5.1. Primary criterion 20
  5.2. Secondary criteria 20
6. IDENTIFICATION OF THE MEDICAL DEVICE 20
7. research design 21
  7.1. Research methodology 21
  7.2. Research process 21
8. NUMBER OF SUBJECTS REQUIRED 24
9. STATISTICAL ANALYSIS 24
10. PROJECT FEASIBILITY 25
11. LOGISTICAL, LEGAL AND GENERAL ASPECTS 25
  11.1. Provisional timetable 25
  11.2. Ethics Committee 25
  11.3. CNIL 25

11.4. Information and no objection 26
11.5. Substantial changes 26
11.6. Data confidentiality 26
11.7. Quality control and assurance 26
11.8. Data collection 27
11.9. Archiving 27
11.10. Insurance 27
11.11. Publication rules 27
12. BIBLIOGRAPHY 29
13. LIST OF APPENDICES 31

SIGNATURE PAGE

PRINCIPAL INVESTIGATOR

I have read every page of the protocol for the clinical trial for which the CHU de Rennes is responsible. I confirm that it contains all the information necessary for the conduct of the trial. I undertake to carry out the trial in compliance with the protocol and the terms and conditions defined therein. I undertake to conduct the trial in accordance with the principles of the "Declaration of Helsinki".

I also undertake to ensure that the investigators and other qualified members of my team will have access to copies of this protocol and the documents relating to the conduct of the trial to enable them to work in accordance with the provisions set out in these documents.

NAME OF PRINCIPAL INVESTIGATOR: **Dr Linda LASSEL**

Signature: ....Date: ....

PERSON IN CHARGE OF THE STUDY

Head of the study: CHU de RENNES

NAME: **Nicolas MEVEL- Head of Research**

Signature: .... Date: ....

SUMMARY

TITLE	Computerized analysis of fetal heart rate variability for early detection of chorioamnionitis in preterm premature rupture of membranes
RESPONSIBLE FOR THE STUDY	Rennes University Hospital
PRINCIPAL INVESTIGATOR	Dr Linda Lassel<br>Department of Gynecology-Obstetrics and Reproductive Medicine<br>Rennes University Hospital<br>Hôpital Sud<br>16 Boulevard de Bulgarie<br>35203 Rennes cedex 2
PROTOCOL VERSION	14.0 of 08 November 2021
JUSTIFICATION/BACKGROUND	Chorioamnionitis is an inflammation or infection of the ovarian cavity, usually of bacterial origin. It occurs secondary to premature rupture of the membranes (PROM) in the majority of cases. PROM is defined as the opening of the water sac twelve hours or more before the onset of labor.<br>Preterm PROM (< 37 weeks' amenorrhea) is a major public health problem. It accounts for 2 to 3% of pregnancies and around a third of premature deliveries, i.e. more than 20,000 births a year in France. In addition to prematurity, the neonatal prognosis in cases of preterm PROM is directly influenced by the presence of chorioamnionitis, estimated to be present in between 15% and 36% of cases, depending on the author.<br>Chorioamnionitis is responsible for an increase in neonatal morbidity and mortality in case of premature delivery. The inflammatory/infectious phenomena induced by chorioamnionitis increase the frequency of respiratory distress, infectious pulmonary pathologies (pneumopathies, alveolitis), necrotizing enterocolitis, periventricular leukomalacia and intracranial hemorrhage, irrespective of prematurity. Chorioamnionitis also increases the risk of white matter disease leading to cerebral palsy. This relationship has been extensively documented *in vitro*, in experimental animal studies and in observational human studies.<br>There are two main definitions of chorioamnionitis in the literature:<br>– histological chorioamnionitis (the most widely accepted definition and used in this research project) corresponds to an inflammatory damage to the placenta (maternal inflammation) and possibly to the umbilical cord (fetal inflammatory reaction) on anatomopathological examination of the placenta.<br>– clinical chorioamnionitis, characterized by the appearance of clinical signs suggestive of inflammation/infection of the uterine cavity
	and/or fetus.

	The strategies used to reduce the risk of chorioamnionitis in cases of preterm PROM are, on one hand, the systematic prescription of antibiotics and, on the other hand, close clinical and biological monitoring to detect its occurrence as early as possible. If necessary, the birth of the child, even if premature, should be induced to avoid worsening the neonatal prognosis by adding the complications of chorioamnionitis to those of prematurity.<br>However, there is currently no specific marker for the early detection of chorioamnionitis:<br>- the clinical signs of chorioamnionitis are variable, non-specific, and usually occur at an advanced stage of infection (uterine contractions, hyperthermia, maternal and/or fetal tachycardia, uterus painful to palpation, foul amniotic fluid).<br>- the positive predictive value of biological markers used in routine clinical practice is very low, particularly for CRP and hyperleukocytosis, which are the most widely used in France. The measurement of certain cytokines seems promising but is still experimental and is not the subject of large series. Furthermore, although the sensitivity of these cytokines is interesting, none of them is specific for fetal or intra-amniotic infection.<br>Thus, in current clinical practice, there is a lack of specific, non-invasive and easily accessible marker(s) for early detection of chorioamnionitis in cases of preterm PROM.<br>Our single-center study carried out at Rennes University Hospital (Appendix 2) on a cohort of 23 patients with preterm PROM showed for the first time that analysis of fetal heart rate (FHR) variability is a promising avenue of research for screening for chorioamnionitis. Indeed, changes in fetal heart rate variability parameters have been observed in cases of histological chorioamnionitis in the context of preterm PROM. These changes included an increase in baseline heart rate ($p=0.02$), an increase in episodes of low variation ($p=0.04$), a decrease in variation at ($p=0.003$) and episodes of high variation ($p < 0.001$) in the last recordings made before birth (spontaneous or induced) in cases of chorioamnionitis. The index of episodes of high variations (ratio of the mean number of episodes of high variation in the last two recordings to the previous four) appears to be a promising tool for the early diagnosis of chorioamnionitis in preterm PROM (sensitivity 90%, specificity 84.6%, positive predictive value 71.5%, negative predictive value 95.2%, area under the ROC curve = 0.88, 95% confidence interval 0.73-100). These data are in line with those observed in cases of neonatal infection and are consistent with the underlying pathophysiological mechanisms (loss of variability,

	reduced fetal adaptability in response to the placental infectious/inflammatory stimulus).<br>In conclusion, early detection of chorioamnionitis in preterm PROM is a major challenge in obstetric research, and an effective diagnostic tool is still lacking. If the results of our pilot study are confirmed, the integration of these markers of cardiac variability into real-time monitoring would meet this objective in a simple, non-invasive and reproducible way.
MAIN OBJECTIVE	The main objective is to measure the diagnostic value of the index of episodes of high variation of the fetal heart rate (ratio between the average of the number of episodes of high variation of the last two recordings and the previous four) for the detection of histologically proven chorioamnionitis in preterm PROM.
SECONDARY OBJECTIVES	The secondary objectives are:<br>- to characterize the evolution of fetal and neonatal heart rate variability parameters (linear and non-linear analysis, see Appendix 1) in a population of preterm PROM with and without histological chorioamnionitis.<br>- to test the hypothesis that the other parameters for analyzing fetal heart rate variability may constitute, alone or in association with, in particular, the index of episodes of high variation, a uni or multivariate indicator of histological chorioamnionitis in preterm PROM.
PRINCIPAL JUDGING CRITERION	The primary criterion is the efficacy indicator (area under ROC curve, sensitivity, specificity, positive predictive value, negative predictive value) of the index of episodes of high variation for the detection of the histological chorioamnionitis in preterm PROM.
SECONDARY JUDGING CRITERIA	- The performance of the approach will also be tested by the changes observed in the parameters of fetal and neonatal heart rate variability (linear and non-linear analysis, see Appendix 1). The quantitative expression (statistical analysis) and modelling (graphical and statistical analysis) of the evolution of these markers will enable us to define their diagnostic value for the diagnosis of histological chorioamnionitis and/or maternal-fetal infection in preterm PROM.<br>- An efficiency indicator (area under the ROC curve, sensitivity, specificity, positive predictive value, negative predictive value) will also be used to assess the diagnostic value of the other heart rate variability analysis parameters selected in the form of either a univariate or multivariate indicator for the detection of histological chorioamnionitis in preterm PROM.

METHODOLOGY / STRUCTURE OF THE STUDY	Monocentric case-control observational study with blinded analysis of nursing staff. Inclusion will be prospective: any pregnant patient with a singleton pregnancy hospitalized for PROM between 26- and 34-weeks' gestation will potentially be included in the study.<br>The Doppler/ECG cardiac signal will be collected prospectively, extracted from recordings made as part of routine monitoring of fetal and neonatal well-being. Analysis of heart rate variability parameters (index of episodes of high variation, linear and non-linear analysis) will be carried out using Matlab® software without the knowledge of the nursing staff. The algorithms for calculating these parameters have already been developed by the Signal and Image Processing Laboratory (LTSI).
INCLUSION CRITERIA	- Adult patient<br>- Singleton pregnancy<br>- PROM occurring between 26- and 34-weeks' gestation, authenticated on clinical examination and, if in doubt, confirmed by a vaginal diagnostic test detecting IGFBP-1.<br>- Patient who has received information about the protocol and has not expressed opposition to participating
NON-INCLUSION CRITERIA	- Multiple pregnancy<br>- Neonatal hypotrophy (birth weight <10th AUDIPOG percentile)<br>- Active maternal smoking<br>- Gestational or pre-pregnancy diabetes<br>- Maternal pathology:<br>o congenital or acquired heart disease.<br>o pulmonary embolism under treatment<br>o pulmonary arterial hypertension<br>o moderate to severe chronic renal failure<br>o chronic obstructive pulmonary disease<br>o autoimmune disease (systemic lupus erythematosus, multiple sclerosis, Gougerot-Sjögren syndrome)<br>- Proven fetal cardiac, neurological or genetic malformation
EXCLUSION CRITERIA FOR THE MAIN OBJECTIVE	Delivery within 48 hours of the onset of PROM (clinical situation not allowing calculation of the index of episodes of high variation or modelling of changes in HRV analysis parameters).
STRATEGY / PRODUCT / MEDICAL DEVICE	Doppler recording of the fetal heart rate signal will be carried out using an F3 Fetal Monitor cardiotocograph (EDAN Instruments, Inc; figure 1), which has the same technical characteristics as all competing cardiotocographs. The advantage of this device is that it has a 60-hour internal memory, enabling recordings to be stored in the form of independent TRC (Trace File) files that can be transferred to a computer via a USB port. These TRC files provide a

	digital record of the fetal heartbeat, resampled to 4 Hz, which can be used to calculate heart rate variability parameters in Matlab®.<br>The neonatal cardiac signal will be recorded by ECG 48 hours after birth, then once a week.
NUMBER OF PATIENTS	120 patients to be included, including 60 patients who could be analyzed for the main objective (calculation based on the results of the pilot study carried out at Rennes University Hospital)
THEORETICAL NUMBER OF CENTRES	4 HUGOPEREN centers (Angers, Nantes, Rennes and Poitiers)
RESEARCH DURATION	Length of inclusion period: 39 months<br>Maximum duration of participation for each patient/newborn pair: an average of 4 weeks of follow-up for the mother, and up to 15 weeks for the newborn, i.e. approximately 19 weeks.<br>Maximum total duration of the study: 50 months
EXPECTED BENEFITS	This project should position the CHU de Rennes as the French and European leader in technological and clinical research for obstetric care.<br>- Medical perspectives: in the long term, to provide clinicians with a decision-support tool by integrating the proposed indicators into a real-time monitoring system that will enable early diagnosis of chorioamnionitis in preterm PROM in a way that is rapid, non-invasive, reproducible and more effective than existing methods. Improving the performance of this diagnosis would lead to a significant reduction in neonatal morbidity and mortality by avoiding adding the complications of chorioamnionitis to those of prematurity in the case of preterm PROM.<br>- Fundamental perspectives: creation of new signal processing tools, understanding the maturation of rhythms during development.<br>- Industrial perspectives: creation of systems to aid in the medical diagnosis of chorioamnionitis and maternal-fetal infection (patent pending), new methods of monitoring and multiparametric functional explorations.<br>- Research perspectives: final evaluation within 2 to 3 years in a multi-center, randomized, intention-to-treat clinical research project (national PHRC); creation of a study network on the variability of cardiac rhythm control and regulation during development.

LIST OF ABBREVIATIONS

ANOVA	ANalysis Of Variance
ASCENT	Anonymised System for Clinical Experimentation
ApEn	Approximate entropy
CRA	Clinical Research Associate
AUDIPOG	Association of Users of Computerised Records in Paediatrics, Obstetrics and Gynaecology
CER	Rennes Ethics Committee
CHU	University Hospital Centre
CIC-IT	Clinical Investigation Centre - Technological Innovation
CNIL	National Commission for Information Technology and Civil Liberties
CRF	Case Report Form
CRP	C-reactive protein
DFA	Detrended Fluctuation Analysis
ECG	Electrocardiography
ETF	Transfontanellar ultrasound
FHR	Fetal heart rate recording
GIRCI	Interregional Group for Clinical Research and Innovation
HF	High frequency
PAH	Pulmonary Arterial Hypertension
IGFBP-1	Insulin-like growth factor-binding protein 1
INSERM	National Institute of Health and Medical Research
MRI	Magnetic Resonance Imaging
LF	Low frequency
LTSI	Signal and Image Processing Laboratory
NICE	National Institute for Health and Care Excellence
NN	Normal to normal
PHRC	Hospital Clinical Research Programme
PNN	Polynuclear neutrophils
FHR	Fetal heart rate
rMSSD	root mean square of successive differences
ROC	Receiver operating characteristic
PROM	Premature rupture of the membranes
WA	Week of amenorrhea
SampEn	Sample entropy
SD	Standard deviation
SDNN	Standard deviation of NN intervals
TRC	Trace file
ULF	Ultra low frequency
UMR	Joint Research Unit

USB	Universal Serial Bus
VCT	Short-term variation
VLF	Very low frequency
HRV	Heart rate variability

1. GENERAL INFORMATION

1.1. Title

Computerized analysis of fetal heart rate variability for early detection of chorioamnionitis in preterm premature rupture of membranes

1.2. Manager

Identity

Rennes University Hospital
2, rue Henri le Guilloux
35033 Rennes Cedex 9

Signature of the protocol on behalf of the person responsible

Pascal GAUDRON - Director of Research
Rennes University Hospital - Pontchaillou Hospital
2, rue Henri le Guilloux
35033 Rennes Cedex 9

Head of research

Pascal GAUDRON - Director of Research
Rennes University Hospital - Pontchaillou Hospital
2, rue Henri le Guilloux
35033 Rennes Cedex 9

1.3. Coordination and monitoring of the study

Research Department
Rennes University Hospital - Pontchaillou Hospital
2, rue Henri le Guilloux
35033 Rennes Cedex 9

1.4. Principal investigator

Dr Linda Lassel
Gynecology and Obstetrics Department
Rennes University Hospital
Hôpital Sud
16 Boulevard de Bulgarie
35203 Rennes cedex 2

1.5. investigator(s)

Dr Christelle Mainguy

Gynecology and Obstetrics Department
Rennes University Hospital
Hôpital Sud
16 Boulevard de Bulgarie
35203 Rennes cedex 2

Dr Pierre-Emmanuel Bouet
Gynecology and Obstetrics Department
Angers University Hospital
4 rue Larrey
49933 Angers Cedex 9

Dr Vincent Dochez
Gynecology and Obstetrics Department
Nantes University Hospital
38 Boulevard Jean Monnet
44093 Nantes Cedex 1

Dr Bertrand GACHON
Gynecology and Obstetrics Department
Poitiers University Hospital
2 Rue de la Milétrie
86021 Poitiers Cedex

1.6. Associate scientists

Professor Guy Carrault
Signal and Image Processing Laboratory (INSERM UMR1099) - SEPIA Team
Campus de Beaulieu, University of Rennes 1
35042 Rennes Cedex

Professor Patrick Pladys
Signal and Image Processing Laboratory (INSERM UMR1099) - SEPIA Team
Campus de Beaulieu, University of Rennes 1
35042 Rennes Cedex

Dr David Riochet
Coordination of the HUGOPEREN pediatric research network in Western France
HME
Nantes University Hospital
44093 Nantes cedex 01

Email : david.riochet@univ-nantes.fr

1.7. Methodologist

Dr Bruno Laviolle, Clinical Investigation Centre, Inserm 1414, Clinical Pharmacology Unit, Pontchaillou Hospital, 2 rue Henri le Guilloux, 35033 Rennes cedex 9. Tel: 02.99.28.96.68 - Email: bruno.laviolle@chu-rennes.fr

2. RATIONALE FOR THE STUDY

Preterm premature rupture of the membranes and early detection of chorioamnionitis

Chorioamnionitis is an inflammation or infection of the ovarian cavity, usually of bacterial origin. It occurs secondary to premature rupture of the membranes (PROM) in the majority of cases (1).

PROM is defined as the opening of the water sac twelve hours or more before the onset of labour. The diagnosis is primarily clinical, with visible discharge of amniotic fluid on clinical examination. In case of doubt, diagnosis is facilitated by the use of tests, of which the most sensitive and specific currently is the detection of IGFBP-1 in vaginal discharge (2).

Preterm PPROM (< 37 weeks' amenorrhea) is a major public health problem. It accounts for 2 to 3% of pregnancies and around a third of premature deliveries, i.e. more than 20,000 births a year in France. (3-5). In addition to prematurity, the neonatal prognosis in cases of preterm PROM is directly influenced by the presence of chorioamnionitis, estimated to be present in between 15 and 36% of cases, according to (6).

Chorioamnionitis is responsible for an increase in neonatal morbidity and mortality in case of premature delivery. The inflammatory/infectious phenomena induced by chorioamnionitis increase the frequency of respiratory distress, infectious pulmonary pathologies (pneumopathies, alveolitis), necrotizing enterocolitis, periventricular leukomalacia and intracranial hemorrhage, regardless of prematurity. (6-8). Chorioamnionitis also increases the risk of white matter disease leading to cerebral palsy. This relationship has been extensively documented *in vitro*, in experimental animal studies and in observational human studies (9-13).

There are two main definitions of chorioamnionitis in the literature:

- the most widely accepted is histological, where pathological examination of the placenta reveals chorionic inflammation (infiltration of neutrophils in the chorionic plate). (1). The associated inflammation of the umbilical vessels (funiculitis), caused by the fetal inflammatory response, is a serious factor. (14). The disadvantage of this definition in current clinical practice is the retrospective nature of the diagnosis.
- clinical chorioamnionitis, characterized by the appearance of clinical signs suggestive of inflammation/infection of the uterine cavity and/or fetus.

After 34 weeks' amenorrhea (WA), there is no consensus on the appropriate course of action to be taken between expectant care with monitoring or a decision to give birth. However, before 34 WA, the management of preterm PROM consists of expectant care with close maternal-fetal monitoring in a high-risk pregnancy unit of a level appropriate to the gestational age. (15). On the other hand, if signs of chorioamnionitis are detected, the pregnancy should be terminated and the baby should be delivered urgently (16).

The strategies used to reduce the risk of chorioamnionitis in cases of preterm PROM are, on one hand, the systematic prescription of antibiotics and, on the other, close clinical and biological monitoring to detect its

occurrence as early as possible.

However, there is currently no specific marker for the early detection of chorioamnionitis:

- the clinical signs of chorioamnionitis are inconstant, variable, non-specific and usually occur at an advanced stage of infection (uterine contractions, hyperthermia, maternal and/or fetal tachycardia, uterus painful to palpation, foul amniotic fluid). (17).
- the positive predictive value of biological markers used in routine clinical practice is very low, particularly for CRP and hyperleukocytosis, which are the most widely used in France. The measurement of certain cytokines seems promising, but is still experimental and is not the subject of large series. Furthermore, although the sensitivity of these cytokines is interesting, none of them is specific for fetal or intra-amniotic infection (18).

A study carried out at Rennes University Hospital (Appendix 2) has shown for the first time that analysis of fetal heart rate (FHR) variability is a promising avenue of research in this indication.

The study of fetal and neonatal heart rate variability: a new avenue of research for the early detection of chorioamnionitis and maternal-fetal infection in preterm PROM

The study of heart rate variability (HRV) provides a non-invasive approach to the activity of the autonomic nervous system. HRV is generally calculated by analyzing the beat-to-beat time series (RR intervals) of ECG.

Various methods of analyzing heart rate variability have been proposed and many parameters have been extracted from them:

- linear analysis, which breaks down into :
    - Time-domain analysis with calculation of SD, rMSSD parameters
    - Frequency-domain analysis, where power is measured in pre-determined bands called LF, HF, VLF
- non-linear analysis where different indices are estimated for example from the Poincaré plot (SD1, SD2) or directly from time series such as the entropy ApEn, SampEn, DFA-α1 (see Appendix 1)

The diagnostic and prognostic applications of HRV studies are numerous, both in adults (sudden death, atherosclerosis, heart failure, sleep apnoea syndrome, diabetic neuropathy) and in adults (sudden cardiac death, atherosclerosis, heart failure, sleep apnoea syndrome, diabetic neuropathy) (19) or in neonatology: sudden infant death syndrome (20) late neonatal infection. It is in fact possible to identify the occurrence of late neonatal infections in premature newborns (over 3 days of life) by analyzing the complexity of the cardiac rhythm and characterising cardiac decelerations. Sepsis in premature newborns is associated with a drop in approximate (ApEn) and sample (SampEn) entropy, a reduction of heart rate variability and transient decelerations (21-24). This monitoring of heart rate characteristics is simple, non-invasive and rapidly available (it requires only 30 minutes of recording of the neonatal electrocardiogram). It is already used routinely in neonatology and has been shown to reduce mortality in low-weight infants (25).

In order to optimize the analysis of fetal HRV and by analogy with the adult and newborn, the acquisition of the fetal ECG non-invasively has been the subject of much work in recent years. This technique is difficult because of the size of the fetal heart and the need for significant signal processing to dissociate the fetal ECG from the maternal ECG. Recent studies have shown that analysis of HRV is only possible on less than 10% of the recording, and is even virtually impossible between 30-34 SA due to the presence of the *vernix caseosa*, which electrically

isolates the fetus, causing significant attenuation of the fetal ECG signal (26). Thus, the most commonly used tool in obstetrics remains the collection of the cardiac signal by ultrasound. Fetal heart rate (FHR) recording is obtained by Doppler by averaging several fetal cardiac cycles.

During pregnancy, the visual study of the fetal HRV is one of the major criteria in the analysis of the FHR, as a first-line examination for assessing fetal well-being, in particular in the monitoring of preterm PROM. Nevertheless, the visual interpretation of the FHR is unreliable, apart from strictly normal rhythms and highly pathological rhythms with numerous decelerations. Several studies have demonstrated the extent of both inter-observer and intra-observer variation in the analysis of the FHR (27,28). A computerized analysis of the FHR, attempting an objective approach to fetal HRV via the measurement of various parameters, has therefore been developed and marketed since 1989 in the form of the OXFORD® 8000 then 8002 (29). It can be used to calculate:

- Basal heart rate: corresponds to the average FHR during the trace, without acceleration or deceleration.
- accelerations and decelerations: deviations from the baseline
- episodes of high and low variation: these episodes are defined as any part of the recording where, relative to the baseline, the variation in amplitude over one minute is greater than 32 ms (high variation) or less than 30 ms (low variation) for 5 to 6 consecutive minutes. Episodes of high variation are associated with active fetal sleep, while episodes of low variation are associated with quiet sleep.
- short-term variation (STV): measures "micro" fluctuations in fetal heart rate and cannot be measured with the naked eye. It is independent of the baseline. STV is measured by dividing each minute of the trace into 16 sections of 3.75 seconds. The average pulse interval of each section is calculated and the change in these average values from one section to the next determines the STV.

Our previous single-center study carried out at the University Hospital of Rennes (Appendix 2, data not yet submitted for publication, pending patent registration) on a cohort of 23 patients with preterm PROM showed for the first time that analysis of the variability of the fetal heart rate (FHR) via Doppler collection of the cardiac signal is a promising avenue of research for screening for chorioamnionitis.

Certain parameters of FHR variability are modified in cases of histologically proven chorioamnionitis. These changes include an increase in baseline heart rate ($p=0.02$), an increase in episodes of low variation ($p=0.04$), a decrease in short-term variation ($p=0.003$) and episodes of high variation ($p < 0.001$) in the last recordings made before birth (spontaneous or induced) in cases of chorioamnionitis. The index of episodes of high variation (ratio of the mean number of episodes of high variation in the last two recordings to the previous four) appears to be a promising tool for the early diagnosis of chorioamnionitis in preterm PROM (sensitivity 90%, specificity 84.6%, positive predictive value 71.5%, negative predictive value 95.2%, area under the curve = 0.88, 95% confidence interval 0.73-100). These data are in line with those observed in cases of neonatal infection and are consistent with the underlying pathophysiological mechanisms (loss of variability, reduced foetal adaptability in response to the placental infectious/inflammatory stimulus).

These results therefore suggest that a prospective study should be carried out to test this index and to confirm the results for all the cardiac signals collected during the monitoring of preterm fetuses.

Conclusion

Early detection of chorioamnionitis in preterm PROM is a major challenge in obstetric research, and an effective diagnostic tool is still lacking. If the results of our pilot study are confirmed, the integration of these markers of HRV into real-time monitoring would meet this objective. Such a simple, easily accessible, reproducible and non-invasive device would provide clinicians with an aid to clinical decision-making (fetal extraction) that would reduce neonatal morbidity/mortality by avoiding adding the complications of chorioamnionitis to those of prematurity at the time of birth in the case of preterm PROM.

3. OBJECTIVES

3.1. Main objective

The main objective is to measure the diagnostic value of the index of episodes of high variation of the fetal heart rate (ratio between the average of the number of episodes of high variation of the last two recordings and the four preceding ones) for the detection of histologically proven chorioamnionitis in preterm PROM.

3.2. Secondary objective(s)

The secondary objectives are:

- to characterize the evolution of fetal and neonatal cardiac variability parameters (linear and non-linear analysis, see Appendix 1) in a population of premature babies with and without histological chorioamnionitis. to test the hypothesis that the other parameters for analysing fetal cardiac variability can constitute, alone or in association, in particular with the index of episodes of high variation, a uni or multivariate indicator of histological chorioamnionitis in preterm premature babies.

4. DEFINITION OF ELIGIBLE SUBJECTS

4.1. Inclusion criteria

The inclusion criteria are:

- adult patient
- singleton pregnancy
- PROM occurring between 26- and 34-weeks' gestation, authenticated on clinical examination and, if in doubt, confirmed by a vaginal diagnostic test detecting IGFBP-1.
- patient who has received information about the protocol and has not expressed opposition to participating.

4.2. Non-inclusion criteria

The criteria for non-inclusion **are:**

- multiple pregnancy
- neonatal hypotrophy (birth weight <10th AUDIPOG percentile)
- active maternal smoking
- gestational or pre-pregnancy diabetes
- maternal pathology:
    - congenital or acquired heart disease

- pulmonary embolism under treatment
- pulmonary arterial hypertension
- moderate to severe chronic renal failure
- Chronic obstructive pulmonary disease
- Autoimmune diseases (systemic lupus erythematosus, multiple sclerosis, Gougerot-Sjögren syndrome)

- fetal malformation with known cardiac, neurological or genetic abnormality

4.3. Exclusion criteria for the main objective

Delivery within 48 hours of PROM (clinical situation not allowing calculation of the index of episodes of high variation or modelling of changes in HRV analysis parameters).

5. JUDGING CRITERIA

5.1. Primary criterion

The primary criterion is the effectiveness indicator (area under the ROC curve, sensitivity, specificity, positive predictive value, negative predictive value) of the index of episodes of high variation for the detection of histological chorioamnionitis in preterm PROM.

5.2. Secondary criteria

- The performance of the approach will also be tested by the changes observed in the parameters of fetal and neonatal cardiac variability (linear and non-linear analysis). The quantitative expression (statistical analysis) and modelling (graphical and statistical analysis) of the evolution of these markers will enable us to define their diagnostic value for the diagnosis of histological chorioamnionitis and/or maternal-fetal infection in preterm PROM.
- an efficiency indicator (area under the ROC curve, sensitivity, specificity, positive predictive value, negative predictive value) will also be used to assess the diagnostic value of the other HRV analysis parameters selected in the form of either a univariate or multivariate indicator for the detection of histological chorioamnionitis in preterm PROM.

6. IDENTIFICATION OF THE MEDICAL DEVICE

Doppler recording of the FHR signal will be carried out using an F3 Fetal Monitor cardiotocograph (EDAN Instruments, Inc; figure 1), which has the same technical features as all competing cardiotocographs. This device has the advantage of being equipped with a 60-hour internal memory, enabling recordings to be stored in the form of independent TRC (Trace File) files, which can be transferred to a computer via a USB port. These TRC files provide a digital record of the fetal heartbeats, resampled at 4 Hz, which can be used to calculate HRV parameters using Matlab® (Mathworks Inc.).

The neonatal cardiac signal will be collected by ECG, 48 hours after birth, then once a week using ECG signal extracts from the polygraph monitor: raw and resampled series at 4 Hz of successive beat-to-beat series (RR), QRS and P morphology axis.

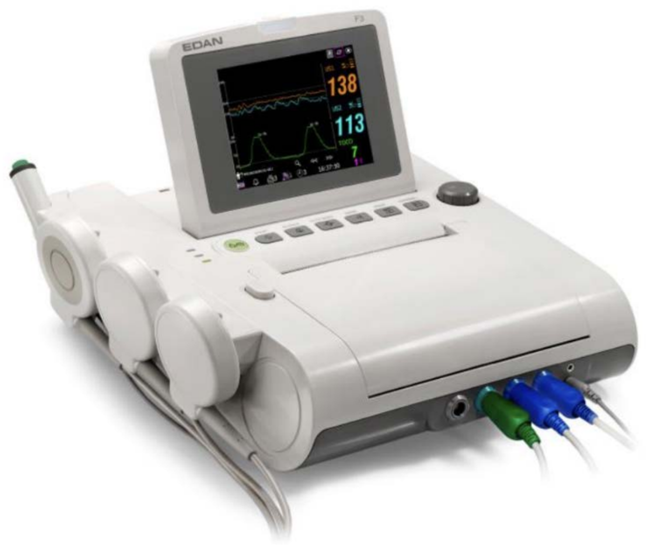

Figure 1: F3 Fetal Monitor cardiotocograph (EDAN Instruments, Inc)

7. RESEARCH DESIGN

7.1. Research methodology

Multicenter observational case-control study with blinded analysis of nursing staff.

7.2. Research process

Inclusion will be prospective: any pregnant patient with a singleton pregnancy hospitalized for PROM between 26- and 34-weeks’ gestation will potentially be included in the study.
Doppler recording of the FHR signal will be prospective from inclusion, as part of the routine of fetal well-being monitoring. Analysis of HRV parameters (index of episodes of high variation, linear and non-linear analysis) will be carried out using Matlab® software without the knowledge of the nursing staff. The algorithms for calculating these parameters have already been developed by the Signal and Image Processing Laboratory.
Clinical data will be collected prospectively in a relational events database. It will cover maternal, placental and neonatal data.
The Doppler data from the FHR signals, anonymized beforehand, will be stored progressively on a computer dedicated to the study. A copy of the signals acquired will be made regularly on the CIC-IT's secure server via the ASCENT system (Anonymized System for Clinical experimentation: https://ascent.univ-rennes1.fr/) and stored progressively on a database dedicated to the study.
In addition, a joint protocol has been drawn up with the investigating centres, setting out in particular the procedures for fetal heart rate recordings. This protocol does not significantly modify the current practices established in these centres, which are already harmonized.

Selection of participants

Patients will be selected from the gynecology and obstetrics departments of the University hospitals of Rennes, Angers, Nantes and Poitiers.

Inclusion visit (=D0)

Its purpose is to present the objectives of the study to the patient, to check the inclusion and non-inclusion criteria and to obtain the patient's non-opposition.

The following data are collected during this visit:

- maternal clinical data
  - Age
  - Height and weight with calculation of body mass index
  - Precise pregnancy dating (obstetric ultrasound performed between 11 WA + 1 day and 14 WA + 6 days)
  - Obstetrical history: parity, previous obstetrical pathology (preterm PROM, preeclampsia, intrauterine growth retardation, premature delivery), mode of delivery (vaginal delivery, instrumental vaginal delivery, caesarean section).
  - Term of premature rupture of the membranes and colour of the amniotic fluid
- fetal data
  - Initial computerized FHR

RPM – birth

The following data is collected during these visits:

- maternal data:
  - biological data: leucocyte counts, CRP assays, results of bacteriological vaginal swabs, clinical data: body temperature, colour of amniotic fluid, etc.
  - date and time of CELESTENE® injections
  - Initiation of tocolysis (ADALATE®, TRACTOCILE®): method of administration, dates and duration of treatment(s)
- Antibiotic therapy: antibiotic(s), dates and duration of treatment(s)
- fetal data:
- FHR: saved on the monitor, transferred weekly via a USB key and sent via the Internet to the Signal and Image Processing Laboratory (LTSI-UMR INSERM 1099) via the secure ASCENT site (https://ascent.univ-rennes1.fr/).
- Oligohydramnios or anamnios

Birth

The following data are collected during this visit:

- mode of delivery and reason (in particular, suspicion of clinical chorioamnionitis) except if spontaneous labour: spontaneous labour, induction, emergency caesarean section
- context of onset of labour: maternal fever, changes in amniotic fluid, uterine contractions, abnormalities in the FHR (visual or computerized)
- Mode of delivery: vaginal delivery, instrumental vaginal delivery, caesarean section

- Birth weight (expressed in absolute value and percentile according to AUDIPOG reference curves)
- Arterial pH and lactates at the umbilical cord, base excess, PCO2,
- How the child is discharged (resuscitation, routine care, intensive care, post-natal care)
- Neonatal death: date, time and autopsy result if performed

Post-natal period (2 days to 15 weeks)

The following data are collected during this period:

- within 72 hours of birth:
    - neonatal biological data: CRP (all), Procalcitonin, peripheral bacteriological samples and blood cultures, blood glucose (random)
    - neonatal clinical data :
        - respiratory: surfactant, ventilation methods
        - cardiovascular: PAH, use of vasopressin amines
        - blood: anaemia (haemoglobin), jaundice (asat, alat, direct and conjugated bilirubin)
        - assessment of suspected maternal-fetal infection: definite, probable or possible according to the criteria of the National Institute for Health and Care Excellence (NICE guidelines CG149)
        - digestive/hepatobiliary: ulcerative enterocolitis with modified Bell stage

    - cardiac variability data: storage of the neonatal ECG (1 recording of one hour in the first 48 hours, then once a week until discharge from hospital)

- remotely:
    - anatomopathological examination of the placenta using a standardized grid to collect information for identifying chorioamnionitis (25,26)
        - acute chorioamnionitis (maternal inflammation)
            - stage 1: infiltrate of neutrophils (PNN) in the roof of the interventricular chamber
            - stage 2: stage 1 + PNN infiltrate in the chorionic plate
            - stage 3: stage 2 + necrosis of PNN and/or amniotic cells
        - foetal inflammatory reaction
            - stage 1: vessels of the chorionic plate and/or umbilical vein
            - stage 2: stage 1 + umbilical artery
            - stage 3: stage 2 + diffusion in Wharton's jelly
        - presence of placental infarcts (old and/or recent) with percentage of placental surface affected (<10%, between 10 and 30%, >30%)
        - presence of a retroplacental hematoma
        - presence of a marginal decidual hematoma
    - assessment of neonatal cerebral pathologies (results of cerebral imaging: ETF and MRI)

Actions	D0<br>(Inclusion visit on admission)	Free interval RPM/birth<br>(RPM at 15 weeks)	Birth	Post-natal period (neonatology followed by a maximum of 15 weeks)
Signature of no objection	X			
Inclusion fax	X			
Maternal-fetal clinical data	X	X		
Collection of computerized FHR	X	X		
Neonatal clinical data			X	X
Pathological examination of the placenta				X
ECG collection			X	X

8. NUMBER OF SUBJECTS REQUIRED

The prevalence of histological chorioamnionitis during preterm PROM is 20 to 30% in the literature (1,10). Given that the mean of the index of high variation episodes (appendix 2, table 1) is in absolute value 3.82 in the case of chorioamnionitis and 1.18 in the absence of chorioamnionitis with a common standard deviation of 1.7, a sample of 60 analysable patients has a power of 95% to detect a significant difference in two-sided formulation and using a Student's t-test with a significant p less than 0.05 based on a prevalence of 15% of histological chorioamnionitis. Given the exclusion criterion for the analysis of the primary objective (delivery within 48 hours of the PROM, which concerns 50% of preterm PROM), a sample of 120 patients is required, 60 of whom can be analyzed for the primary objective.

Taking into account a safety margin of 30% (refusal to participate or presence of non-inclusion criteria), this study requires a minimum potential inclusion of 160 patients.

The planned recruitment is realistic. The annual numbers of pre-term POM at the CHU de Rennes, Angers, Nantes and Poitiers can be estimated at 100, 100, 100 and 60 respectively.

9. STATISTICAL ANALYSIS

The predictive capacity of the index of episodes of high variation and the uni/multivariate indicator obtained from HRV parameters will be estimated on the basis of sensitivity, specificity, false-positive and false-negative rates, and the area under the ROC curve.

An exploratory univariate analysis of the parameters used to analyse HRV will be carried out. Comparisons of the distributions, means and medians of these parameters in the monitoring blocks associated with histological chorioamnionitis and in the blocks not associated with histological chorioamnionitis will be carried out (Student's *t* test and Mann-Whitney test).

Changes over time in the various parameters measured will be compared between the two populations using a 2-factor repeated time ANOVA (gestational age and chorioamnionitis). The subject effect will be included in the model to take account of the matched nature of the measurements.

10. PROJECT FEASIBILITY

The feasibility of this project is based on a privileged environment combining the complementary skills of several entities:

1/ The **Signal and Image Processing Laboratory (LTSI - UMR INSERM 1099),** which provides knowledge of signal collection, processing and analysis, as well as long-standing expertise in the control and development of medical monitoring systems. The work carried out by the SEPIA team at the LTSI on the detection of infection in very premature babies is currently being evaluated as part of the Caress-Premi PHRC, and will form a solid basis for the success of this project.

With the support of the Groupement Interrégional de Recherche Clinique et d'Innovation des Hôpitaux Universitaires (GIRCI) du Grand Ouest (PhysioDev project), the LTSI is also behind the design and development of the ASCENT secure anonymisation and data storage system (https://ascent.univ-rennes1.fr/), which guarantees the transmission of FHR data under optimum conditions.

2/ This project benefits from the support of the **HUGOPEREN network** (Hôpitaux Universitaires du Grand Ouest PEdiatric REsearch Network), financed by the GIRCI du Grand Ouest, with the **Gynecology-Obstetrics** departments **of the CHU of Rennes** (>4000 deliveries/year potential for inclusion : 100 preterm PROM/year), **Angers** (>4,000 deliveries/year, potential inclusion: 100 preterm PROM/year), **Nantes** (>4,000 deliveries/year, potential inclusion: 100 preterm PROM/year) and **Poitiers** (>2,500 deliveries/year, potential inclusion: 60 preterm PROM/year), which guarantee the recruitment required for this study.

3/ The **Centre d'Investigation Clinique et d'Innovation Technologique (CIC 1414) at Rennes University Hospital,** which is providing its technical facilities and expertise in validating signal processing algorithms and medical devices.

11. LOGISTICAL, LEGAL AND GENERAL ASPECTS

11.1. Provisional timetable

Length of inclusion period: 39 months

Maximum duration of participation for each mother/baby pair: approximately 19 weeks (5 months)

Duration of data processing and reports: 6 months

Maximum total duration of the study: 50 months

11.2. Ethics Committee

Before any research is carried out, the study leader, CHU de Rennes, will submit the study protocol and the letter of information and non-objection to the Rennes Ethics Committee for its opinion.

11.3. CNIL

This study falls within the scope of the "Reference Methodology" (MR-003) in application of the provisions of

article 54 paragraph 5 of law no. 78-17 of 6 January 1978 as amended relating to information technology, files and civil liberties. The CHU de Rennes, promoter of the study, has signed a commitment to comply with this "Reference Methodology".

11.4. Information and no objection

Patients will be fully and fairly informed, in comprehensible terms, of the objectives of the study, their right to refuse to take part in the study and the possibility of withdrawing their consent at any time, including for their child. All this information will be included in an information and non-objection letter given to patients.
All or part of the data collected as part of this research protocol may be transferred to other researchers in France or abroad in so far as the subject has not exercised his/her right to object.

11.5. Substantial changes

Any substantial modification to the study protocol must be notified to the competent authorities.

11.6. Data confidentiality

Persons with direct access will take all necessary precautions to ensure the confidentiality of information relating to the persons concerned, in particular as regards their identity and the results obtained.
These people, in the same way as the investigators themselves, are subject to professional secrecy (under the conditions defined by articles 226-13 and 226-14 of the French Penal Code).
During or at the end of the research, the data collected on the persons involved and transmitted to the person in charge of the study by the investigators (or any other specialist) will be rendered anonymous.
Under no circumstances may the names or addresses of the persons concerned appear in clear text.
Only the first letter of the subject's surname and first name will be recorded, together with a coded number specific to the study indicating the order of inclusion of subjects.

11.7. Quality control and assurance

A Clinical Research Associate (CRA) appointed by the head of the study will ensure that the study is carried out properly, that the data generated are collected in writing, and that they are documented, recorded and reported in accordance with the legislative and regulatory provisions in force relating to the conduct of a non-interventional study.
Each investigator and the members of his/her team agree to make themselves available for Quality Control visits carried out at regular intervals by the Clinical Research Associate. During these visits, the following elements will be reviewed:
- follow-up of inclusions
- compliance with the study protocol and the procedures defined therein.

The investigators undertake to accept any quality assurance audits carried out by the study leader and any

inspections carried out by the Competent Authorities. All data, documents and reports may be the subject of audits and regulatory inspections without being subject to medical confidentiality.

11.8. Data collection

All the information required by the protocol must be recorded in the observation books. Data should be collected as it is obtained and recorded explicitly in the notebooks. Any missing data should be coded.
This electronic observation book will be set up in each of the centres using an Internet data collection medium. Investigators will be provided with a document to help them use this tool.
If the investigator fills in the case report form via the Internet, the CRA can view the data quickly and remotely. The investigator is responsible for the accuracy, quality and relevance of all data entered. In addition, when data is entered, it is immediately checked for consistency. In this respect, he must validate any change in value in the CRF. These changes are subject to an audit trail. A justification may be included as a comment. A paper printout will be requested at the end of the study, authenticated (dated and signed) by the investigator. A copy of the authenticated document sent to the sponsor must be archived by the investigator.

The Doppler data from the FHR signals and the ECG recordings, which have been anonymized beforehand, will be stored progressively on a computer dedicated to the study. A copy of the acquired signals will be made weekly via USB port from the monitors, then transmitted to the CIC-IT's secure server via the ASCENT (Anonymized System for Clinical experimentation: https://ascent.univ-rennes1.fr/) device and stored progressively on a database located on a computer dedicated specifically to the study.

11.9. Archiving

The following documents will be kept in the respective departments until the end of the period of practical use. These documents are :

- Protocol and annexes, any substantial amendments,
- Individual data (authenticated copies of raw data)
- Follow-up documents
- Statistical analysis
- Final study report

Study documents must be archived by the study manager for a minimum of 15 years.
Nothing may be moved or destroyed without the agreement of the person in charge of the study. At the end of the 15-year period, the person in charge of the study will be consulted for destruction. All data, documents and reports may be audited or inspected.

11.10. Insurance

The CHU of Rennes is responsible for the study, and there is no need to take out insurance for this non-interventional study.

11.11. Publication rules

The rules for publication are as follows:

- signed by the project leader and then by those who make a significant contribution during the course of the study.
- the source of the funding.

In the case of ancillary studies, the results of these may only be published with the agreement of the project leader and only after publication of the main study, which must be cited.

12. BIBLIOGRAPHY

1 Redline RW, Faye-Petersen O, Heller D, Qureshi F, Savell V, Vogler C, et al. Amniotic infection syndrome: nosology and reproducibility of placental reaction patterns. Pediatr Dev Pathol. 2003 Sep;6(5):435-48.

2 Gallot D, Guibourdenche J, Sapin V, Goffinet F, Doret M, Langer B, et al. Which biological test to confirm rupture of membranes? J Gynecol Obstet Biol Reprod. 2012 Apr;41(2):115-21.

3 Parry S, Strauss JF. Premature rupture of the fetal membranes. N Engl J Med. 1998 Mar 5;338(10):663-70.

4 Simhan HN, Canavan TP. Preterm premature rupture of membranes: diagnosis, evaluation and management strategies. BJOG. 2005 Mar;112 Suppl 1:32-7.

5 Goldenberg RL, Culhane JF, Iams JD, Romero R. Epidemiology and causes of preterm birth. Lancet. 2008 Jan 5;371(9606):75-84.

6 Aziz N, Cheng YW, Caughey AB. Neonatal outcomes in the setting of preterm premature rupture of membranes complicated by chorioamnionitis. J Matern Fetal Neonatal Med. 2009 Sep;22(9):780-4.

7 Ramsey PS, Lieman JM, Brumfield CG, Carlo W. Chorioamnionitis increases neonatal morbidity in pregnancies complicated by preterm premature rupture of membranes. Am J Obstet Gynecol. 2005 Apr;192(4):1162-6.

8 Dammann O, Leviton A, Gappa M, Dammann CEL. Lung and brain damage in preterm newborns, and their association with gestational age, prematurity subgroup, infection/inflammation and long term outcome. BJOG. 2005 Mar;112 Suppl 1(s1):4-9.

9 Wu YW. Systematic review of chorioamnionitis and cerebral palsy. Ment Retard Dev Disabil Res Rev. 2002;8(1):25-9.

10 Yoon BH, Romero R, Park JS, Kim CJ, Kim SH, Choi JH, et al. Fetal exposure to an intra-amniotic inflammation and the development of cerebral palsy at the age of three years. Am J Obstet Gynecol. 2000 Mar;182(3):675-81.

11 Spinillo A, Capuzzo E, Stronati M, Ometto A, Orcesi S, Fazzi E. Effect of preterm premature rupture of membranes on neurodevelopmental outcome: follow up at two years of age. BJOG. 1995;102(11):882-7.

12 Dammann O, Kuban KCK, Leviton A. Perinatal infection, fetal inflammatory response, white matter damage, and cognitive limitations in children born preterm. Ment Retard Dev Disabil Res Rev. 2002;8(1):46-50.

13 Yoon BH, Park C-W, Chaiworapongsa T. Intrauterine infection and the development of cerebral palsy. BJOG. 2003 Apr;110 Suppl 20:124-7.

14 Pacora P, Chaiworapongsa T, Maymon E, Kim YM, Gomez R, Yoon BH, et al. Funisitis and chorionic vasculitis: the histological counterpart of the fetal inflammatory response syndrome. J Matern Fetal Neonatal Med. 2002 Jan;11(1):18-25.

15 Collège des Gynécologues et Obstétriciens Français (CNGOF). Rupture prématurée des membranes: recommandations pour la pratique clinique. wwwcngofassofr. (1999).

16 American College of Obstetricians and Gynecologists. Practice Bulletins No. 139. Obstet Gynecol. 2013 Oct;122(4):918-30

17 Audibert F. Diagnosis of infection in the case of premature rupture of the membranes. J Gynecol Obstet

Biol Reprod. 1999 Nov;28(7):635-41

18 Popowski T, Goffinet F, Batteux F, Maillard F, Kayem G. Prediction of maternofetal infection in preterm premature rupture of membranes: serum maternal markers. Gynecol Obstet Fertil. 2011 May;39(5):302-8.

19. Xhyheri B, Manfrini O, Mazzolini M, Pizzi C, Bugiardini R. Heart rate variability today. Prog Cardiovasc Dis. 2012 Nov;55(3):321-31.

20 Pincus SM, Cummins TR, Haddad GG. Heart rate control in normal and aborted-SIDS infants. Am J Physiol. 1993 Mar;264(3 Pt 2):R638-46.

21 Griffin MP, O'Shea TM, Bissonette EA, Harrell FE, Lake DE, Moorman JR. Abnormal heart rate characteristics are associated with neonatal mortality. Pediatr Res. 2004 May;55(5):782-8.

22 Griffin MP, O'Shea TM, Bissonette EA, Harrell FE, Lake DE, Moorman JR. Abnormal heart rate characteristics preceding neonatal sepsis and sepsis-like illness. Pediatr Res. 2003 Jun;53(6):920-6.

23 Griffin MP, Lake DE, Bissonette EA, Harrell FE, O'Shea TM, Moorman JR. Heart rate characteristics: novel physiomarkers to predict neonatal infection and death. Pediatrics. 2005 Nov;116(5):1070-4.

24 Beuchée A, Carrault G, Bansard JY, Boutaric E, Bétrémieux P, Pladys P. Uncorrelated randomness of the heart rate is associated with sepsis in sick premature infants. Neonatology. 2009;96(2):109–14.

25 Moorman JR, Carlo WA, Kattwinkel J, Schelonka RL, Porcelli PJ, Navarrete CT, et al. Mortality reduction by heart rate characteristic monitoring in very low birth weight neonates: a randomized trial. J Pediatr. 2011 Dec;159(6):900-1.

26. van Laar JOEH, Warmerdam GJJ, Verdurmen KMJ, Vullings R, Peters CHL, Houterman S, et al. Fetal heart rate variability during pregnancy, obtained from non-invasive electrocardiogram recordings. Acta Obstet Gynecol Scand. 2013 Oct 17;93(1):n/a-n/a.

27 Bernardes J, Costa-Pereira A, Ayres-de-Campos D, van Geijn HP, Pereira-Leite L. Evaluation of interobserver agreement of cardiotocograms. Int J Gynaecol Obstet. 1997 Apr;57(1):33-7.

28 Gagnon R, Campbell MK, Hunse C. A comparison between visual and computer analysis of antepartum fetal heart rate tracings. Am J Obstet Gynecol. 1993 Mar;168(3 Pt 1):842-7.

29 Dawes GS, Moulden M, Redman CW. System 8000: computerized antenatal FHR analysis. J Perinat Med. 1991;19(1-2):47-51.

13. LIST OF APPENDICES

Appendix 1: Analysis of cardiac variability

Appendix 2: Computerized analysis of fetal heart rate and early detection of chorioamnionitis in premature rupture of membranes (summary of the retrospective study carried out at Rennes University Hospital)

Appendix 3: Information and no-objection letter

Appendix 4: Joint management protocol
