# Supplementary material for: Day-to-day dynamics of fetal heart rate variability to detect chorioamnionitis in preterm premature rupture of membranes
Source: PLoS One. 2025 Jan 2;20(1):e0305875. doi: 10.1371/journal.pone.0305875 (PMC11695014; doi:10.1371/journal.pone.0305875)
Supplement: S1 Table — Features with significant differences are indicated with * and shown in bold. (PDF) [file pone.0305875.s004.pdf]

Table S4. Comparison between chorioamnionitis and non-chorioamnionitis populations for all the extracted features. Features with significant differences are indicated with * and shown in bold.

Type of features	Feature	Non-Chorioamnionitis (Stage 0)	Chorioamnionitis (Stage 1 & 2 & 3)	p-value
**cFHR**	baseline FHR	142.77 ± 7.33 (128.44; 137.89; 142.30; 146.70; 161.88)	143.79 ± 9.03 (116.92; 138.88; 143.60; 148.77; 166.04)	0.1939
	Accelerations nb/min	0.07 ± 0.06 (0; 0.03; 0.06; 0.10; 0.24)	0.05 ± 0.05 (0; 0; 0.03; 0.07; 0.264106)	**0.0101 ***
	Accelerations duration/min	20.67 ± 11.76 (0; 18.25; 21.4; 27.125; 45.375)	16.88 ± 12.14 (0; 0; 19.75; 25.75; 42)	0.0515
	Accelerations size/min	0.41 ± 0.48 (0; 0.11; 0.29; 0.52; 2.99)	0.27 ± 0.33 (0; 0; 0.17; 0.40; 1.76)	**0.0056 ***
	Decelerations nb/min	0.001 ± 0.004 (0; 0; 0; 0; 0.03)	0.0006 ± 0.004 (0; 0; 0; 0; 0.03)	0.5714
	Decelerations duration/min	0.8 ± 5.19 (0; 0; 0; 0; 35)	0.57 ± 4.95 (0; 0; 0; 0; 43)	0.5775
	Decelerations size/min	0.01 ± 0.07 (0; 0; 0; 0; 0.56)	0.02 ± 0.20 (0; 0; 0; 0; 2.44)	0.5714
	LTV	61.35 ± 14.21 (29.27; 50.83; 60.51; 70.42; 95.12)	55.76 ± 16.42 (29.79; 44.98; 52.13; 64.79; 141.72)	**0.0008 ***
	STV	4.50 ± 1.13 (2.20; 3.71; 4.38; 5.21; 7.73)	4.04 ± 1.23 (2.11; 3.15; 3.75; 4.81; 8.74)	**0.0006 ***
	EpHV nb/min	0.71 ± 0.19 (0.10; 0.63; 0.78; 0.86; 0.92)	0.63 ± 0.21 (0.02; 0.50; 0.67; 0.80; 0.93)	**0.0022 ***
	EpHV duration/min	0.86 ± 0.18 (0.22; 0.82; 0.93; 0.99; 1)	0.80 ± 0.20 (0.12; 0.69; 0.88; 0.97; 1)	**0.0075 ***
	EpLV nb/min	0.05 ± 0.11 (0; 0; 0; 0.04; 0.60)	0.07 ± 0.11 (0; 0; 0; 0.13; 0.44)	0.05660
	EpLV duration/min	0.08 ± 0.16 (0; 0; 0; 0.10; 0.70)	0.12 ± 0.17 (0; 0; 0; 0.21; 0.75)	0.0512

**Time-FHRV**	mean	422.20 ± 21.34<br>(371.20; 409.37; 422.37; 435.60; 469.12)	419.68 ± 27.12<br>(361.84; 403.91; 418.37; 432.43; 514.89)	0.1767
	SD	18.66 ± 4.75<br>(9.33; 15.22; 18.17; 20.85; 38.81)	17.13 ± 5.97<br>(8.94; 13.85; 15.71; 19.64; 64.60)	**0.0017 ***
	maximum	520.60 ± 40.87<br>(450.32; 494.27; 518.43; 543.96; 688.47)	516.63 ± 58.85<br>(408.54; 480.86; 509.04; 537.87; 905.78)	0.1408
	minimum	360.17 ± 20.89<br>(307.68; 347.20; 361; 372.68; 402.91)	363.79 ± 22.90<br>(308.96; 349.68; 363.40; 373.61; 434.07)	0.3845
	RMSSD	7.43 ± 1.84<br>(3.93; 6.13; 7.35; 8.46; 13.02)	6.62 ± 2.10<br>(3.39; 5.08; 6.51; 7.49; 14.31)	**0.0003 ***
	SDSD	7.43 ± 1.84<br>(3.93; 6.13; 7.35; 8.46; 13.02)	6.616407 ± 2.10<br>(3.39; 5.08; 6.51; 7.50; 14.31)	**0.0003 ***
	kurtosis	6.87 ± 5.55<br>(2.73; 4.18; 5.43; 6.64; 34.23)	7.61 ± 7.53<br>(3.01; 4.07; 5.35; 8.48; 70.48)	0.4089
	skewness	0.48 ± 0.99<br>(-1.14; -0.15; 0.31; 0.81; 3.63)	0.62 ± 1.01<br>(-1.10; -0.10; 0.42; 1.06; 6.05)	0.2183
**Frequency-FHRV**	LF	162000000 ± 84506155<br>(48318581; 103000000; 146000000; 197000000; 453000000)	141000000 ± 126000000<br>(29294785; 81515985; 112000000; 157000000; 1290000000)	**0.0024 ***
	HF	11387962 ± 7216711<br>(2272070; 6994465; 9740177; 12932332; 42780420)	9421836 ± 7285103<br>(1905824; 4223709; 7753967; 11439320; 42644796)	**0.0025 ***
	LFHF	16.50 ± 7.43<br>(4.54; 10.5; 15.72; 20.58; 33.82)	17.75 ± 9.19<br>(4.23; 11.03; 16.71; 22.98; 48.18)	0.5458
	LFnu	0.93 ± 0.03<br>(0.82; 0.91; 0.94; 0.95; 0.97)	0.93 ± 0.03<br>(0.81; 0.92; 0.94; 0.96; 0.98)	0.5471
	HFnu	0.07 ± 0.03<br>(0.03; 0.05; 0.06; 0.09; 0.18)	0.07 ± 0.03<br>(0.02; 0.04; 0.06; 0.08; 0.19)	0.5471
**Nonlinear-FHRV**	ApproxEntropy	1.05 ± 0.19<br>(0.45; 0.91; 1.01; 1.18; 1.47)	1.01 ± 0.18<br>(0.43; 0.88; 1.00; 1.13; 1.51)	0.1519

	sampEntropy	0.80 ± 0.21<br>(0.29; 0.65; 0.75; 0.97; 1.30)	0.77 ± 0.19<br>(0.23; 0.65; 0.74; 0.88; 1.31)	0.5877
	SD1	5.26 ± 1.30<br>(2.78; 4.33; 5.20; 5.98; 9.21)	4.68 ± 1.48<br>(2.40; 3.59; 4.61; 5.30; 10.12)	**0.0003 ***
	SD2	25.83 ± 6.69<br>(12.86; 21.07; 25.13; 29.19; 54.52)	23.76 ± 8.38<br>(12.38; 19.13; 21.80; 27.41; 90.82)	**0.0023 ***
	DFA-alfa1	1.26 ± 0.10<br>(1.03; 1.20; 1.26; 1.32; 1.53)	1.28 ± 0.10<br>(0.96; 1.22; 1.27; 1.33; 1.59)	0.1273
	DFA-alfa2	0.30 ± 0.08<br>(0.15; 0.24; 0.28; 0.35; 0.54)	0.28 ± 0.09<br>(0.12; 0.22; 0.27; 0.34; 0.56)	0.0861
	AC	1.48 ± 0.51<br>(0.58; 1.12; 1.40; 1.75; 2.95)	1.31 ± 0.53<br>(0.40; 0.92; 1.16; 1.65; 3.58)	**0.0053 ***
	DC	1.64 ± 0.60<br>(0.65; 1.20; 1.58; 1.93; 3.60)	1.42 ± 0.57<br>(0.47; 0.99; 1.27; 1.85; 3.53)	**0.0047 ***

Data are represented as mean ± standard deviation (minimum ; 25th percentile ; median; 75th percentile ; maximum)
